# Human Fetal Brain-Derived Neural Stem/Progenitor Cells Grafted into the Adult Epileptic Brain Restrain Seizures in Rat Models of Temporal Lobe Epilepsy

**DOI:** 10.1371/journal.pone.0104092

**Published:** 2014-08-08

**Authors:** Haejin Lee, Seokhwan Yun, Il-Sun Kim, Il-Shin Lee, Jeong Eun Shin, Soo Chul Park, Won-Joo Kim, Kook In Park

**Affiliations:** 1 Brain Korea 21 Plus Project for Medical Science, Yonsei University College of Medicine, Seoul, Korea; 2 Severance Children's Hospital, Department of Pediatrics, Yonsei University College of Medicine, Seoul, Korea; 3 Department of Neurology, Yonsei University College of Medicine, Seoul, Korea; Université Pierre et Marie Curie-Paris6, INSERM, CNRS, France

## Abstract

Cell transplantation has been suggested as an alternative therapy for temporal lobe epilepsy (TLE) because this can suppress spontaneous recurrent seizures in animal models. To evaluate the therapeutic potential of human neural stem/progenitor cells (huNSPCs) for treating TLE, we transplanted huNSPCs, derived from an aborted fetal telencephalon at 13 weeks of gestation and expanded in culture as neurospheres over a long time period, into the epileptic hippocampus of fully kindled and pilocarpine-treated adult rats exhibiting TLE. *In vitro*, huNSPCs not only produced all three central nervous system neural cell types, but also differentiated into ganglionic eminences-derived γ-aminobutyric acid (GABA)-ergic interneurons and released GABA in response to the depolarization induced by a high K^+^ medium. NSPC grafting reduced behavioral seizure duration, afterdischarge duration on electroencephalograms, and seizure stage in the kindling model, as well as the frequency and the duration of spontaneous recurrent motor seizures in pilocarpine-induced animals. However, NSPC grafting neither improved spatial learning or memory function in pilocarpine-treated animals. Following transplantation, grafted cells showed extensive migration around the injection site, robust engraftment, and long-term survival, along with differentiation into β-tubulin III^+^ neurons (∼34%), APC-CC1^+^ oligodendrocytes (∼28%), and GFAP^+^ astrocytes (∼8%). Furthermore, among donor-derived cells, ∼24% produced GABA. Additionally, to explain the effect of seizure suppression after NSPC grafting, we examined the anticonvulsant glial cell-derived neurotrophic factor (GDNF) levels in host hippocampal astrocytes and mossy fiber sprouting into the supragranular layer of the dentate gyrus in the epileptic brain. Grafted cells restored the expression of GDNF in host astrocytes but did not reverse the mossy fiber sprouting, eliminating the latter as potential mechanism. These results suggest that human fetal brain-derived NSPCs possess some therapeutic effect for TLE treatments although further studies to both increase the yield of NSPC grafts-derived functionally integrated GABAergic neurons and improve cognitive deficits are still needed.

## Introduction

Epilepsy is a chronic neurological disorder affecting tens of millions of people worldwide, and more than 30% of patients with epilepsy still have uncontrolled seizures despite conventional antiepileptic drugs (AEDs) [1]. Temporal lobe epilepsy (TLE) is the most frequent and pharmaco-resistant type of adult focal epilepsy that presents with complex partial seizures, originating mainly from the mesial temporal structures such as the hippocampus or amygdala [2], [3]. Loss of γ-aminobutyric acid (GABA)-ergic inhibition and aberrant synaptic reorganization changes have been observed in the hippocampus of patients with TLE and animal models [4]–[6], resulting in an increase in the overall neuronal excitatory tone compared with inhibitory function [2], [7]. Although surgical removal of epileptic foci is a recommended treatment for patients with TLE, this therapeutic option can lead to undesirable complications, such as significant cognitive impairment and lasting dependence on AEDs [8]. For this reason, a continuing need exists for novel approaches that effectively control seizures in chronic TLE.

Stem cell-based cell transplantation has been investigated intensively for epilepsy treatment in various animal studies, although none of these approaches has yet been tested clinically in patients with epilepsy [9]–[11]. Over the past few years, embryonic stem cell (ES)-, neural stem cell (NSC)-, or neural precursor-based approaches have been examined in animal models of epilepsy: mouse ES-derived neural precursors in pilocarpine- or kainic acid-induced status epilepticus (SE) or kindling-based TLE models [12]–[15], rat fetal ganglionic eminence (GE)-derived neural precursors or NSC in the kainic acid-induced TLE model [16], [17], mouse fetal neural precursors from the medial ganglionic eminence (MGE) in congenital general epilepsy or pilocarpine-induced adult TLE models [18], [19], and immortalized human fetal brain-derived NSC in the pilocarpine-induced SE model [20]. These studies have demonstrated that neural stem/progenitor cell (NSPC)-based therapies in acute and chronic models of epilepsy exert anticonvulsant and antiepileptogenic effects, and may replace degenerated or ablated neurons and repair damaged neural circuitry [7], [9]–[11].

Prior to the clinical application of NSPCs for epilepsy treatment, many challenges still must be addressed. First, it is essential to study human NSPCs (huNSPCs) derived from various cell sources, such as developing and adult brain tissues, ES, and induced pluripotent stem cells (iPSCs), for their abilities in terms of engraftment, migration, differentiation into specific neuronal or glial cells, seizure control, and functional recovery following transplantation into the brains of TLE models [21].

A previous study demonstrated that substantial reductions in spontaneous recurrent motor seizures (SRMSs) were observed for a short-term period after immortalized human fetal NSPCs were infused into the tail vein of the animals a day following pilocarpine-induced SE [20]. However, that study was not pertinent for treating patients with TLE.

Because patients with epilepsy refractory to AED treatment are much in need of novel seizure-suppressing therapy, it is significant to examine the effects of huNSPCs transplantation in animals with established epilepsy at the time of grafting. Previously, we cultivated and expanded several types of huNSPCs that were isolated from different brain regions of an aborted fetus at 13 weeks of gestation as neurospheres in culture dishes [22]. Among them, telencephalon-derived NSPCs gave rise to not only neuronal and glial cells, but also differentiated into more neurons and GABA^+^ cells than other brain region-derived NSPCs in vitro.

The kindling model is one of the most commonly used animal models of TLE. The advantages of kindling are that specific regions of the brain can be stimulated in a controlled fashion [5], [23]. However, in the kindling model, seizures are evoked and SRMSs are scarce. The pilocarpine-induced model of TLE is characterized by robust and frequent SRMSs, behavioral abnormalities, and poor responses to AEDs [2], [5], [24]. These animal models develop several typical lesions that appear to be analogous to those in human TLE.

Given this background, we investigated whether epileptic phenotypes could be improved in both kindling and pilocarpine-induced TLE models by human fetal brain-derived NSPC grafts into the hippocampus after epileptic seizures emerged, and characterized the distribution, engraftment, and the differentiation patterns of cells implanted in adult recipients. Grafted cells showed extensive migration, robust engraftment, long-term survival, differentiation into three central nervous system (CNS) neural cell types, and provided a large number of GABAergic interneurons around the implanted sites. Moreover cell grafting has therapeutic potentials for treating TLE, particularly with regard to seizure suppression. However, further studies to both increase the yield of NSPC grafts-derived functionally integrated GABAergic neurons and improve cognitive deficits are still needed.

## Materials and Methods

### Cell culture

Human fetal brain tissue from a cadaver at 13 weeks of gestation was obtained with full parental written consent and approval of the Research Ethics Committee of Yonsei University College of Medicine, Seoul, Korea (Permit Number: 4-2003-0078) [25]. In this study, huNSPCs for transplantation were derived from such a single donated fetal tissue. The culture of NSPCs was previously described in detail [22]. Briefly, after dissociation of telencephalic tissue in trypsin (0.1% for 30 min; Sigma, St. Louis, MO), cells were plated at 4×10^5^ cells/mL in serum-free culture medium (DMEM/F12; Gibco, Grand Island, NY), N2 formulation (1% v/v; Gibco), and 8 µg/mL heparin (Sigma) supplemented with 20 ng/mL fibroblast growth factor-2 (FGF-2; R&D Systems, Minneapolis, MN) and 10 ng/mL leukemia inhibitory factor (LIF; Sigma). All cultures were maintained in a humidified incubator at 37°C and 5% CO_2_ in air, and half of the growth medium was changed every 3–4 days. Proliferating single cells in culture generated free-floating neurospheres during the first 2–5 days of growth. They were passaged every 7–8 days by dissociation of bulk neurospheres with 0.05% trypsin/EDTA (T/E; Gibco).

For proliferation conditions, 8×10^5^ cells/well were maintained with mitogens in 6-well plates for quantitative RT-PCR, Western blot and HPLC analysis. For differentiation conditions, neurospheres were trypsinized and dissociated into single cells, and cells were then placed on poly-L-lysine (PLL; 10 µg/mL; Sigma)-coated 8-well chamber slides (Nunc, Roskilde, Denmark) at 8×10^4^ cells/well for immunocytochemical analysis or on PLL-coated 6-well plates (Sigma) at 1×10^6^ cells/well for quantitative RT-PCR, Western blot and HPLC analysis in the growth medium without mitogens. The medium was replaced every 2 days, and cells were analyzed at day 7 of differentiation.

### Quantitative real-time reverse transcription-polymerase chain reaction (qRT-PCR)

Total RNA was isolated from cell cultures using the TRI reagent (MRC, Inc., Cincinnati, OH), and 2 µg of RNA was reverse-transcribed into cDNA using random hexamer primers (Bioneer, Daejeon, Korea) and Superscript III reverse transcriptase (Invitrogen, Grand Island, NY) in a thermal cycler (Eppendorf, Happauge, NY) according to the manufacturer's instructions. Quantitative RT-PCR was carried out in a total volume of 10 µL containing 5 µL of LightCycler® 480 SYBR Green I Master (Roche Diagnostics Ltd., Rotkreuz, Switzerland), 0.5 µM of each primer and 2.5 µL of 1∶10 diluted cDNA using LightCycler® 480 instrument (Roche Diagnostics Ltd). The cycling conditions were: 95°C for 5 min, followed by 45 cycles of 95°C for 10 s, 60°C for 10 s, and 72°C for 10 s. All the samples were carried out in triplicates. The expression levels of each mRNA expression were normalized to the housekeeping gene GAPDH using LightCycler® 480 Software, Version 1.5 (Roche Diagnostics Ltd). Primer sequences for *FOXG1*, *DLX2*, *NKX2.1*, *GAD1*, calbindin2 (*CALB2*) and somatostatin (*SST*) have been previously described [26]. Other primer sequences were retrieved from PrimerBank Database http://pga.mgh.harvard.edu/primerbank/ [27], with the exception of *SLC32A1* which was designed using ProbeFinder software Version 2.49 from Roche Applied Science. Sequences of primer sets were listed in Table S1.

### Western blots

At the protein level, the expression of GAD65 (GAD2) and GAD67 (GAD1) was quantified by Western blotting. Cells were lysed in tissue protein extraction reagent (T-PER reagent; Pierce, Rockford, IL) with a protease inhibitor cocktail (Sigma), and lysates were centrifuged (16,000×g, 30 min, 4°C). The supernatant was collected and stored at –80°C. The samples were assayed for total protein using a bicinchoninic acid assay (BCA kit; Pierce), and 20 µg of protein was mixed with 2% β-mercaptoethanol in tricine sample buffer (Bio-Rad, Hercules, CA) and denatured by heating to 95°C. Then, samples (20 µL) were loaded onto 10% Tris–glycine gels, and the proteins were transferred from the gel onto a 0.45-µm nitrocellulose membrane (Thermo Scientific, Suwanee, GA) over 4 h at 4°C. The protein blots were blocked with 5% skimmed milk in TBST and then incubated with 0.25% bovine serum albumin in TBST overnight at 4°C with the following primary antibodies: anti-GAD65 (1∶1,000; Sigma), anti-GAD67 (1∶1,000; Chemicon, Temecula, CA), or β-actin (1∶2,000; Sigma). Immunoblots were rinsed with TBST, incubated with a horseradish peroxidase-conjugated secondary antibody (1∶20,000; Jackson Immunoresearch, West Grove, PA) for 1 h at room temperature, and developed using SuperSignal West Pico Chemiluminescent substrate (1∶20,000; Thermo Scientific). The images were scanned with a Fujifilm LAS-4000 mini imager and analyzed with the MultiGauge software (Fujifilm, Tokyo, Japan).

### High-performance liquid chromatography (HPLC)

Intracellular GABA content was measured 7 days after plating as follows. The cells under proliferation or differentiation conditions were washed twice with ice-cold Hank's balanced salt solution-HEPES (H-H) buffer (Gibco), treated with 0.4 M perchloric acid, and scraped from each well. The cell lysates were centrifuged (16,000×*g*, 4°C, 30 min), and the supernatant was collected and adjusted to pH 7–8. The samples were filtered and stored at −80°C. To measure the level of GABA release, the cells were incubated in basal medium (144 mM NaCl, 1 mM MgCl_2_, 4 mM KCl, 1.8 mM CaCl_2_, 5 mM glucose, and 10 mM HEPES) and high K^+^ medium (same as basal medium except 94 mM NaCl and 53 mM KCl) for 60 min. After incubation, the supernatant was collected, filtered, and stored at −80°C. The GABA levels in the cell lysates and supernatants from basal and high K^+^ media were analyzed by reverse-phase HPLC using *o*-phthalaldehyde derivatization with a fluorescence detector [28], [29]. All experiments were done in triplicate. Values were normalized to total cellular protein, measured using the BCA assay.

### Immunostaining

For immunocytochemical analysis, cultured cells were fixed with 4% paraformaldehyde (PF) in PIPES buffer (Sigma), rinsed with phosphate-buffered saline (PBS) solution, blocked with 3% bovine serum albumin (Sigma), 10% normal horse serum, and 0.3% Triton X-100 (Sigma) in PBS. For immunohistochemical analysis, animals were deeply anesthetized with ketamine (75 mg/kg, i.p.) and xylazine (30 mg/kg, i.p.) and perfused with 4% PF in 0.1 M PIPES buffer. Brains were then removed, post-fixed, transferred in 30% sucrose in PBS for cryoprotection, and frozen in O.C.T compound (Sakura Finetek, Torrance, CA, USA). The brains were coronally sliced into 16-µm sections using a cryostat. Sections were washed in PBS and blocked as mentioned above. Cultures or Sections were incubated with following primary antibodies: anti-human specific nestin (anti-hNestin; 1∶200; Chemicon), anti-human specific nuclear pore (hNP; 1∶20; Oncogene, Cambridge, MA), anti-human specific cytoplasm SC121 (1∶500; Stem Cells, Inc., Cambridge, UK), anti-human specific GFAP SC123 (1∶500; Stem Cells, Inc., Cambridge, UK), anti-glial fibrillary acidic protein (GFAP; 1∶1,000; Dako, Glostrup, Denmark), anti-neuronal class β-tubulin III (TUJ1; 1∶1,000, Covance, Princeton, NJ), anti-GABA (1∶500; Sigma), anti-calretinin (1∶2,000; Chemicon), anti-GAD65 (1∶250; Sigma), anti-GAD67 (1∶500; Chemicon), anti-platelet-derived growth factor receptor alpha (PDGFR-α; 1∶100; Santa Cruz Biotechnology, Santa Cruz, CA), anti-adenomatous polyposis coli-CC1 (APC-CC1; 1∶30; Abcam, Cambridge, MA), anti-Olig2 (1∶500; Millipore, Billerica, MA), anti-glial cell-derived neurotrophic factor (GDNF; 1∶50; Santa Cruz Biotechnology), anti-S100β (1∶1000; Sigma), and anti-human specific nuclei (hNuc; 1∶100; Chemicon) antibodies. Species-specific secondary antibodies, conjugated with fluorescein (FITC; 1∶180; Vector, Burlingame, CA) or Texas Red (TR; 1∶180; Vector) were used to detect the binding of primary antibodies. Specimens were mounted using Vectashield mounting medium with 4,6-diamino-2-phenylindole (DAPI; Vector), and were analyzed by an immunofluorescence microscopy (BX51; Olympus, Center Valley, PA) or a confocal laser scanning microscopy (LSM 700; Carl Zeiss, Oberkochen, Germany).

### Quantification

For *in vitro* quantification, the total number of TUJ1-, PDGFR-α-, GFAP-, GABA-, CALB2-, and GAD2-expressing cells were counted in three to five randomly selected fields. The percentage of each marker-positive cell among total DAPI-positive cells was calculated. This sampling was replicated three times.

To quantify the survival and migration of engrafted huNSPCs, the number of BrdU^+^ cells were quantified in every fifth section through the entire anteroposterior extent of grafts at 4 and 8 weeks after grafting in the kindling model. Estimates of total BrdU^+^ cell numbers from raw cell counts were corrected according to the Abercrombie formula [30]. The mean yield of surviving cells in each rat was depicted as the percentage of transplanted cells. Cell numbers versus distance to the injection site were quantified and values were expressed as a percentage of the total number of surviving cells. To determine differentiation patterns of grafted cells, sets of 50–100 BrdU^+^ or hNuc^+^ cells from every fifth section were used to calculate the percentages of TUJ1^+^, GFAP^+^, APC-CC1^+^, PDGFR^+^, or GABA^+^ cells at 8 weeks following transplantation.

### Kindling process

Adult male Sprague–Dawley rats (∼300 g) were kept on a 12/12-h light/dark cycle (lights on at 07:00 h) with free access to food and water. This study was performed in strict accordance with the recommendations in the Guide for the Care and Use of Laboratory Animals of the National Institute of Health. The protocol was approved by the Committee on the Ethics of Animal Experiments of Yonsei University College of Medicine (Permit Number: 2010-0201). The rats were anesthetized with ketamine (75 mg/kg, i.p.) and xylazine (30 mg/kg, i.p.) and implanted with a bipolar electrode-cannula guide (Plastics One, Roanoke, VA) into the right hippocampal CA3 using the following stereotaxic coordinates: 4.2 mm lateral, 4.52 mm caudal, and 4.5 mm ventral to bregma. The electrode-cannula consisted of a cylindrical plastic pedestal molded around a piece of stainless steel tubing, which guided an infusion cannula, and two individually insulated wire electrodes that attached to the sides of the stainless steel tubing [31]. Via the stainless steel tubing, the infusion cannula penetrated to a consistent depth and was used to transfer vehicle or dissociated NSPCs. Two steel screws served as ground electrodes. All electrodes were anchored in place with dental cement. After a recovery period of 1 week, afterdischarge threshold (ADT) was determined by passing constant pulse trains (1 ms, biphasic rectangular wave, 50 Hz for 1 s). The initial current of 10 µA was delivered, increasing the current intensity in steps of 10 µA (to a maximum of 150 µA), with intervals of 1 min between current deliveries until an afterdischarge (AD) was seen, defined as poststimulus electroencephalogram (EEG) spikes with a frequency of greater than 1 Hz and an amplitude at least two times greater that of the pre-stimulus recording [32]. The rats were stimulated at the ADT intensity twice daily until five consecutive stage 5 or higher seizures (according to the additional Racine scale [33]) were elicited: Stage 1, facial movements only; Stage 2, facial movements and head nodding; Stage 3, facial movements, head nodding, and forelimb clonus; Stage 4, facial movements, head nodding, forelimb clonus, and rearing; Stage 5, facial movements, head nodding, forelimb clonus, rearing, and falling; Stage 6, cluster of multiple Stage 5 seizures; Stage 7, jumping and running seizures; and Stage 8, Stage 7 plus tonic hindlimb extension and tail rigidity, sometimes culminating in death. In total, 25 fully hippocampal-kindled rats were randomly allocated to the two groups: the vehicle-injected control group (*n* = 13) or the NSPC-transplanted group (*n* = 12). The average ADT between the groups was not significantly different (47.7±11.4 and 44.1±7.0 µA in the vehicle and NSPCs groups, respectively; *P* = 0.80).

For fully hippocampal-kindled animals, electrical responses generally consisted of three epochs: primary AD, silent period, and subsequent secondary AD [34]. AD duration (ADD) refers to the cumulative duration of primary and secondary ADs recorded within ∼2–3 min after stimulation at the intensity of ADT. In addition to ADD, behavior seizure stage and seizure duration were measured. Seizure stage was classified according to the additional Racine scale. Seizure duration was defined as the period of limbic seizure (stages 1–2) and subsequent motor seizure (stages 3–7), excluding limbic seizure after motor seizure [35].

### Induction of status epilepticus by pilocarpine

Adult male Sprague–Dawley rats (200–220 g) were infused with lithium chloride (127 mg/kg, i.p.; Sigma) 24 h prior to the administration of pilocarpine. On the next day, the rats were injected with methylscopolamine bromide (1 mg/kg, i.p.; Sigma) to limit the peripheral effects of pilocarpine, and 30 min later were injected with pilocarpine hydrochloride (45 mg/kg, i.p.; Sigma) to induce SE. Seizure events in pilocarpine-treated rats were scored according to the additional Racine scale [33], and rats that did not develop stage 5 or higher seizure were excluded from this study. Diazepam (Samjin, 10 mg/kg, i.p.) was injected 1 h after SE onset to cease seizure activity. The rats that went into SE were injected with 2.5 mL of 5% dextrose intraperitoneally twice a day and were given a moistened rat diet during the following 2–3 days. For 14–20 d after SE, pilocarpine-treated rats were monitored to confirm the emergence of SRMSs by video recording. The frequency of SRMSs (stages 3–7 seizure) was scored for 12 h/day. After monitoring, epileptic rats were randomly allocated to two groups: the vehicle-injected control group (*n* = 18) or the NSPC-transplanted group (*n* = 21). The frequency of SRMSs before transplantation between two groups was not significantly different (0.18±0.04 and 0.15±0.02 seizures/day in the vehicle and NSPCs groups, respectively; *P* = 0.87).

### Preparation and transplantation of NSPCs

huNSPCs were maintained by passaging through the dissociation of bulk neurospheres and cryopreserved at each passage in the Good Manufacturing Practice facility. For transplantation, NSPCs taken at between passage number 10 (P10) and P20 were selected and prepared. Cells were labeled with bromodeoxyuridine (BrdU; 3 µM; Sigma) for 5 days before grafts. At the time of grafting, cells were harvested by trypsinization after which the enzymatic activity was halted by soybean trypsin inhibitor (Sigma). The cells were centrifuged (900×*g*, 3 min), the cell pellet was washed three times with H-H buffer, and the entire cell pellet was then resuspended in H-H buffer at a density of 1.0×10^5^ cells/µL. The concentrated NSPCs in a sterile freezing tube (Nunc) were then delivered to the animal operation room. For the kindling model, 1 week after reaching the kindling criteria, rats were anesthetized and injected with 4 µL of vehicle (H-H buffer only) or NSPC suspension into the stimulated site (CA3 region of the right hippocampus) using an infusion cannula and tubing (Plastics One). For the pilocarpine model, at 3 weeks after SE, epileptic rats were anesthetized and injected with 4 µL of vehicle or NSPC suspension into the CA3 regions of bilateral hippocampus using the following stereotaxic coordinates: 4.2 mm left and right lateral, 4.52 mm caudal, and 5.0 mm ventral to bregma. Vehicle or NSPC suspension was infused at a flow rate of 1 µL/min using a 10-µL Hamilton syringe placed on an infusion pump (KD Scientific, Holliston, MA) controlled by a microprocessor. All animals in both groups received daily injections of cyclosporine (10 mg/kg, i.p.) from a day before transplantation to the end of the experiment.

### Evaluation of NSPC grafts on kindled seizures and SRMS

For the kindling model, after a recovery period of 1 week following transplantation, the hippocampus of each animal was stimulated weekly at ADT intensity. Regular determinations of seizure parameters—ADD, seizure duration, and seizure stage—in both vehicle-injected and NSPC-transplanted groups were performed weekly for 8 weeks with video and EEG recordings. For the pilocarpine model, the behavior of epileptic rats was observed at 1–3 months following transplantation to analyze the frequency, severity and duration of SRMSs in both the vehicle-injected and NSPC-transplanted groups. Starting at 2 weeks following transplantation, the rats were video-monitored for 60 h per week (12 h/day, 5 days/week, 2 weeks/month, and 360 h in total). Rats were given free access to water and food in individual cages and video-monitored during the daylight period. The video recordings were analyzed by observers blinded to group allocation. Rats were scored according to the same additional Racine scale used in the establishment of the kindled model [33]. The frequency and severity of seizure, and the total time spent in seizure were assessed in each rat. At the end of experiments, all rats were sacrificed by CO_2_ inhalation, in accordance with institutional and AVMA guidelines for the euthanasia [36].

### Morris water maze

The Morris water maze test was performed to assess the effect of huNSPCs grafts on learning and memory function in epileptic rats. The detailed method has been previously described [37]. Briefly, a circular polypropylene pool (200 cm in diameter and 40 cm in height) was filled with water (22±1°C) made opaque with a food coloring agent, rendering it impossible for rats to see through it. On the pool rim, four points were designated (north, east, south, west), dividing the pool into four quadrants (NE, NW, SW, SE). A circular platform (15 cm in diameter and 30 cm in height) was positioned at the center of the SE quadrant and hidden 1 cm below the water surface, and the position of the platform was kept unchanged throughout the training days. A trial started when the rat was positioned in the water from a quasirandom start points and ended when the rat reached and escaped onto the hidden platform. The swim paths of rats were recorded by a video tracking system (SMART; Panlab, Barcelona, Spain) and analyzed thereafter. For the pilocarpine model, the water maze test was performed 3 months following transplantation in an age-matched intact group (*n* = 6), the vehicle-injected group (*n* = 11), and the NSPC-transplanted group (*n* = 12). Rats were trained for 4 days, and the training day consisted of six trials with an interval of 5–15 min. If the rat was not able to find the platform in 120 s, the rat was guided to it by the investigator. Mean escape latency was calculated for each day during the training days. At the probe trial, on day 5, the rats were positioned in the opposite quadrant where the platform was previously located and allowed to swim in the pool without the platform for 60 s. All parameters of memory retention were measured. At the end of experiments, all rats were sacrificed by CO_2_ inhalation as described above.

### Determination of GDNF-positive astrocytes in the hippocampus

To investigate whether huNSPC grafting affects the level of GDNF expression in host hippocampal astrocytes, double immunofluorescence for GDNF and the astrocytic marker, S100β was done 12 weeks after transplantation in age-matched intact controls and pilocarpine-treated rats that received vehicle or NSPCs. The ratio of double-labeled GDNF- and S100β-positive cells among total S100β-positive cells in the bilateral CA3 regions of the hippocampus was determined by a confocal laser scanning microscopy in every 10^th^ section throughout the dorsal hippocampus. At least 80 S100β-positive cells per animal were analyzed at 400x magnification.

### Timm staining

After completion of the Morris water maze, pilocarpine-induced epileptic rats were anesthetized with ketamine (75 mg/kg, i.p.) and xylazine (30 mg/kg, i.p.) and perfused transcardially for 10 s with 0.1% sodium sulfide, 3 min with 3% glutaraldehyde in 0.1 M phosphate buffer (pH 7.4), and 7 min with 0.1% sodium sulfide [38]. The brains were postfixed for 24 h at 4°C and then cryoprotected in 30% sucrose in 0.1 M phosphate buffer. The brains were embedded in O.C.T compound (Sakura Finetek), coronally sectioned at 16-µm, and stored at −20°C. A 1-in-10 series of sections from dorsal hippocampus was dried, and developed for 60 min in a solution of 50% gum Arabic (120 mL), 20 ml of 2 M citrate buffer, 3.4 g of hydroquinone in 60 mL of H_2_O, and 1 mL of 17% silver nitrate [39]. After rinsing, sections were dehydrated in graded alcohol and mounted with Permount (Fisher Scientific, Fair Lawn, NJ). Sections from age-matched intact control rats were stained at the same time as sections from pilocarpine-induced epileptic rats. Mossy fiber sprouting was assessed by scoring a five-point scale, as previously reported [40]. The Timm score of each section was calculated by an observer blinded to group allocation, and averaged from all the stained sections of bilateral hippocampi.

### Statistical analysis

All data are shown as means ± standard error of the mean (SEM). Differences in GABA content or GABA release from huNSPCs were evaluated with the Mann–Whitney *U*-test. Significant differences in average yield of viable huNSPCs and between 4 and 8 weeks after transplantation in the kindling model were analyzed with Student's *t*-test. To compare the mean rostrocaudal distance to injection site of grafted NSPCs between 4 and 8 weeks post-transplantation, we used Student's *t*-test. The Timm scores were analyzed using one-way analysis of variance (ANOVA) followed by the *post hoc* Bonferroni test. Comparison of percentages of S100β^+^ astrocytes expressing GDNF in the CA3 region of the hippocampus among the three groups was performed by one-way ANOVA with LSD *post hoc* test. In the kindling model, Student's *t*-test was used to compare the effects of NSPC transplantation with vehicle injection on ADD, seizure duration, and seizure stage. Escape latency in the Morris water maze was compared among groups using repeated-measures ANOVA with Tukey's HSD *post hoc* procedure. Dwell time in the target quadrant, dwell time in the platform area, latency to the platform area, and platform area crossings in the Morris water maze were compared among groups using one-way ANOVA followed by the *post hoc* LSD test. In the pilocarpine model, Student's *t*-test was used to compare the effects of NSPC transplantation with vehicle injection on the frequency, severity and duration of SRMSs. Differences were considered statistically significant for *P*<0.05. Group sizes were calculated based on power analysis (PASS program). We used a power of 0.8 and significance levels of 0.05.

## Results

### Multipotent human NSPCs and differentiation into GABAergic neurons in culture

**Figure 1:**
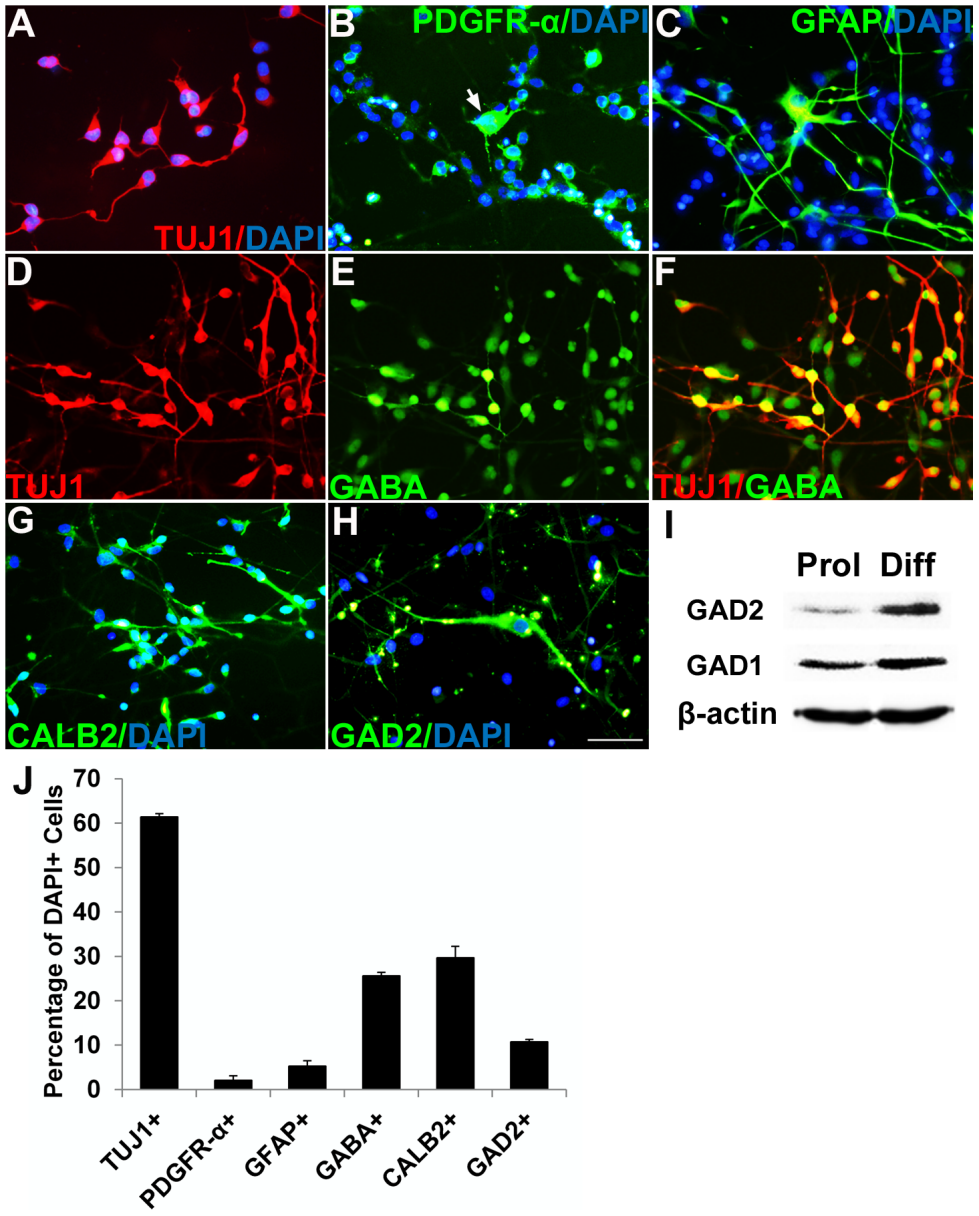
Multipotent human NSPCs and differentiation into GABAergic neurons in culture. (A–C) Under differentiation conditions, differentiation of fractions of NSPCs into TUJ1^+^ neurons visualized by Texas Red (A), PDGFR-α^+^ oligodendrocyte progenitors identified by fluorescein (B), and GFAP^+^ astrocytes imaged using fluorescein (C) could be observed. Nuclei were counterstained with DAPI. (D–F) Many NSPC-derived differentiated neurons were co-labeled with TUJ1 (red) and GABA (green) with small bipolar processes. (G, H) Fractions of NSPC-derived cells also expressed CALB2 (calbindin2, green) and GAD2 (GAD65, green). Scale bar, 50 µm. (I) Western blot analysis shows that GAD1 and GAD2 were expressed highly in NSPCs under differentiation conditions. (J) The bar chart illustrates percentages of NSPCs that exhibit differentiation into TUJ1^+^ neurons, PDGFR-α^+^ oligodendrocyte progenitors, GFAP^+^ astrocytes, GABA^+^ neurons, CALB2^+^ neurons, and GAD2^+^ neurons. Quantification of the data presented in (J); mean ± SEM (*n* = 3).

huNSPCs have the ability to differentiate into all three neural cell types in vitro: neurons, oligodendrocytes, and astrocytes (Fig. 1A–C). At 7 days after plating of neurosphere-derived single cells under differentiation conditions, ∼61% of NSPCs had differentiated into TUJ1^+^ neurons, ∼2% into PDGFR-α^+^ oligodendrocyte progenitors, and ∼5% into GFAP^+^ astrocytes (Fig. 1J). To investigate whether NSPCs could differentiate into GABAergic interneurons, we examined the expression of GABAergic neuronal markers in NSPCs using Western blots and immunocytochemistry (Fig. 1D-J). Histological analysis showed that ∼26% of NSPC-derived differentiated cells expressed GABA (Fig. 1E, J), and ∼37% of NSPC-derived TUJ1^+^ neurons expressed GABA with small bipolar processes (Fig. 1D–F, J). Additionally, ∼30% of cells expressed interneuron subtype marker CALB2, also known as calretinin [26] (Fig. 1G, J), and ∼11% of cells expressed GABA-synthesizing enzyme, GAD2 (Fig. 1H, J). Western blot showed that protein levels of GAD1 and GAD2, the two isoforms of the GABA-synthesizing enzyme, were elevated markedly in cells under differentiation (Diff) conditions, versus proliferation (Prol) conditions (Fig. 1I).

**Figure 2:**
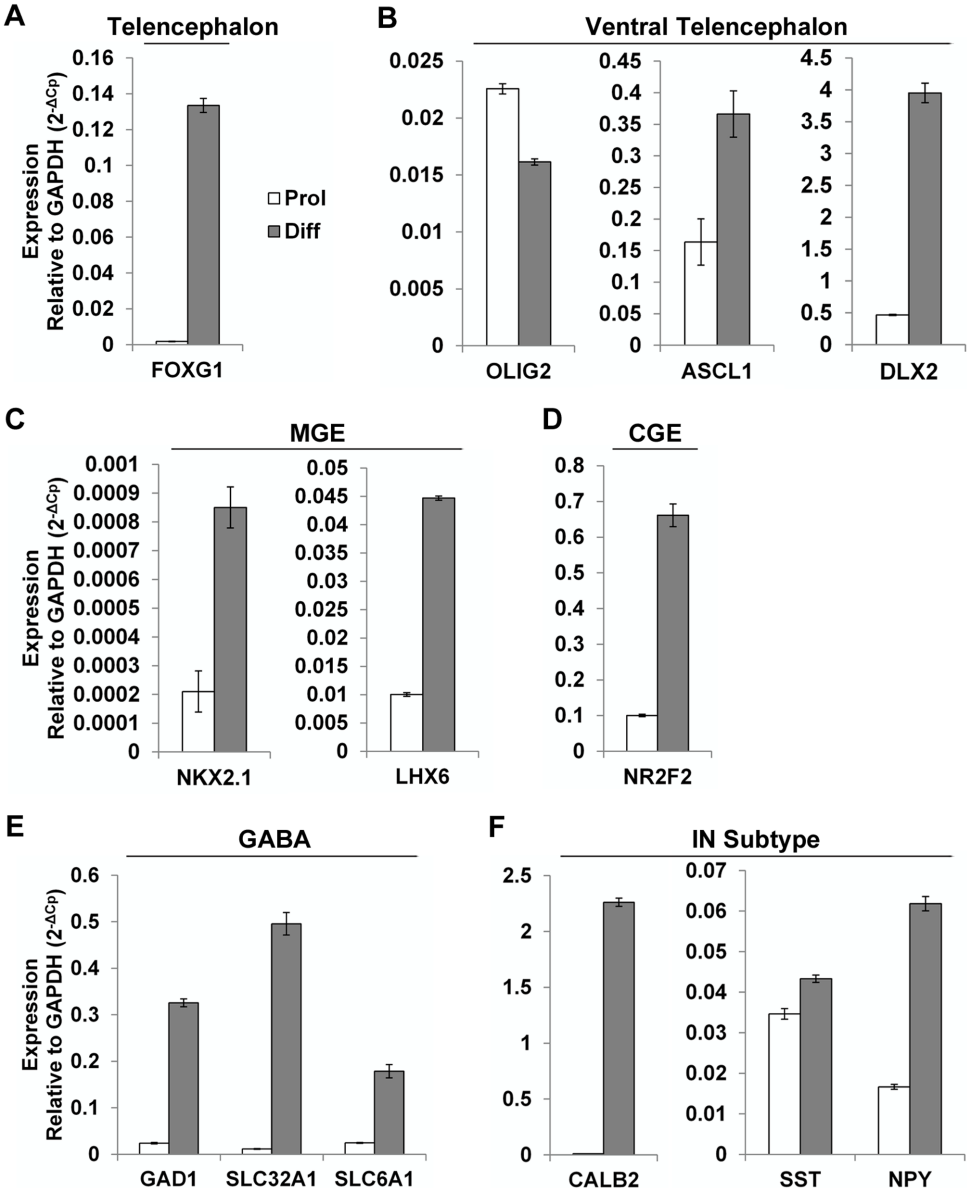
Transcript expression profiling of human NSPCs. (A–F) Expression patterns of genes associated with development and function of GABAergic neurons were analyzed in NSPCs under proliferation (Prol) and differentiation conditions (Diff) using quantitative RT-PCR. The expression levels of each mRNA expression were normalized to levels of GAPDH. Panels show: telencephalic (A), ventral telencephalic GABAergic neuronal lineage (B), medial ganglionic eminence (MGE) (C), caudal ganglionic eminence (CGE) (D), GABAergic neuron (E), and interneuron (IN) subtype markers (F). The expression levels of makers were significantly elevated under Diff condition compared to under Prol condition except *OLIG2*. Data represented as mean ± SEM (n = 3).

We analyzed transcript expression for region-specific and GABAergic interneuron lineage markers in huNSPCs using qRT-PCR (Fig. 2). Telencephalic marker *FOXG1* and ventral telencephalic markers (*OLIG2*, *ASCL1*, and *DLX2*) were expressed in cells under both Prol and Diff conditions [26], [41], and the expression levels of makers were significantly elevated under Diff condition compared to under Prol condition except *OLIG2* (Fig. 2A, B). The MGE, marked by *NKX2.1* and *LHX6* expression, is known the primary source of cortical interneurons both in rodents and in humans, and the caudal ganglionic eminence (CGE), marked by *NR2F2* (*COUP-TFII*) expression, give rise to a greater proportion of cortical interneurons in humans than in rodents [26], [42]-[45]. We found that NSPCs not only expressed MGE markers, but also abundantly the CGE marker (Fig. 2C, D). In addition, GABAergic markers (*GAD1*, *SLC32A1*, and *SLC6A1*) and interneuron subtype markers (*CALB2*, *SST*, and *NPY*) were robustly expressed under differentiation conditions (Fig. 2E, F). These data demonstrate that huNSPCs not only give rise to all three CNS neural cell types, but also that many of them differentiate into the MGE and CGE-derived GABAergic interneurons.

### HPLC analysis of GABA release from human NSPCs in culture

**Figure 3:**
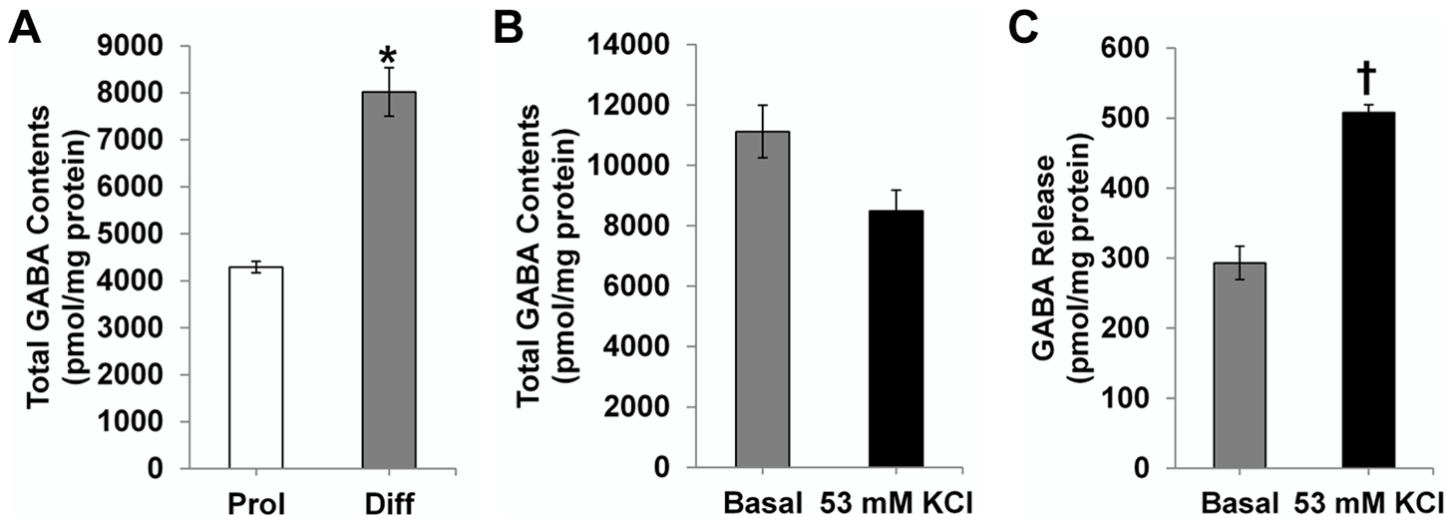
HPLC analysis for GABA in human NSPCs. (A) huNSPCs contain GABA under both proliferation (Prol) and differentiation (Diff) conditions in culture. Note that the total intracellular GABA content of NSPCs was significantly higher under Diff conditions than under Prol conditions. (B, C) NSPCs under Diff conditions were incubated in basal (4 mM KCl) or high K^+^ (53 mM KCl) medium, and intracellular GABA content (B) and GABA release into the medium (C) were quantified. * Significantly different from that under Prol conditions at *P*<0.05; † significantly different from that in the basal medium at *P*<0.05; error bars indicate ±SEM.

After finding evidence that huNSPCs could differentiate into GABAergic neurons, we examined whether NSPC-derived cells actually released GABA under basal and high K^+^ conditions in culture using HPLC. Intracellular GABA content of NSPCs under differentiation conditions (8,018.4±514.9 pmol/mg, *n* = 3) was about twice higher than that under proliferation conditions (4,288.4±118.4 pmol/mg) (*P* = 0.010; Fig. 3A). When NSPCs under differentiation conditions were incubated in basal or high K^+^ medium, the intracellular GABA content of cells incubated in basal medium (11,119.8±871.8 pmol/mg, *n* = 3) was slightly higher than that in the high K^+^ medium (8,494.0±682.0 pmol/mg) (*P* = 0.20; Fig. 3B). However, the amount of GABA released from differentiated NSPCs into the medium was significantly higher in the high K^+^ medium (507.69±11.39 pmol/mg) than that in the basal medium (293.20±23.78 pmol/mg) (*P* = 0.029; Fig. 3C), suggesting that huNSPC-derived differentiated cells can increase efflux of GABA in response to depolarization induced by elevated K^+^.

### Engraftment and distribution of human NSPCs following transplantation

**Figure 4:**
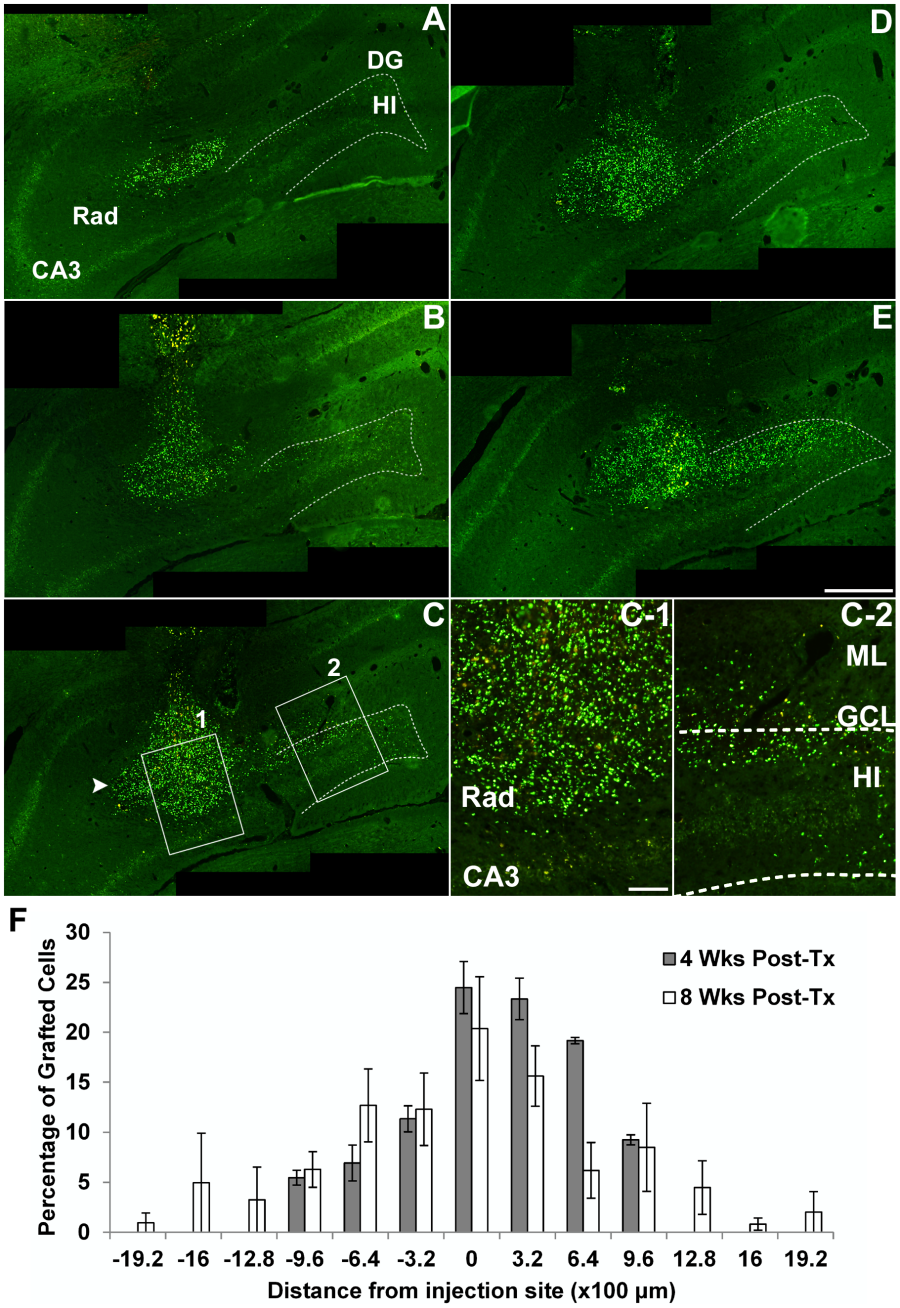
Engraftment and distribution of human NSPCs into the hippocampus of kindled rats. (A–E) Serial sections (320 µm apart) throughout the hippocampus were used to determine the location of BrdU^+^ grafted cells, visualized with fluorescein at 4 weeks following transplantation. A–E panels are representative images of serial coronal sections of the hippocampus which are ordered from anterior to posterior. Grafted cells had migrated apart from the injection site (arrowhead in C) and dispersed throughout the adjacent hippocampal subfields. Many grafted cells were predominantly placed in the radiatum layer (Rad) of the CA3 region, molecular (ML) and granular cell layer (GCL) of the dentate gyrus (DG), and hilus (HI) of the hippocampus. Dotted lines denote the boundary between HI and GCL of the DG. In panel C, the boxed regions of the Rad (1), and ML, GCL and HI (2) are magnified in panel C-1 and C-2, respectively. Scale bar, 500 µm (A–E) and 100 µm (C-1, C-2). (F) The bar chart illustrates the distribution of grafted cells versus rostrocaudal distances to injection site at 4 and 8 weeks after transplantation. The number of grafted cells/distance of serial sections was expressed as a percentage of the total number of surviving cells. Values are represented as mean ± SEM.

In the kindling model, we transplanted NSPCs into the CA3 region of the right hippocampus of fully kindled rats. To evaluate the grafted cells, animals were killed at 4 and 8 weeks following transplantation, and brain tissues were processed for immunohistochemistry. BrdU^+^ grafted cells had migrated away from the injection site and dispersed throughout the hippocampus. Many grafted cells were predominantly located in the radiatum layer of the CA3 region, molecular and granular layer of the dentate gyrus (DG), and hilus of the hippocampus (Fig. 4A-F).

Quantification of the total number of BrdU^+^ cells demonstrated that the survival rate of grafted cells was 57.4±16.6% (229,469.1±66,242.0, *n* = 4 rats) and 29.3±10.0% (117,127.8±39,809.3, *n* = 4) at 4 and 8 weeks following transplantation, respectively. To evaluate the migratory ability of NSPCs, we quantified the number of grafted cells versus rostrocaudal distances to injection site at 4 and 8 weeks after transplantation (*n* = 4, respectively). Grafted cells showed to migrate from the injection site in both rostral and caudal directions. There was a significant difference between the mean distance of migration at 8 weeks and that at 4 weeks (1.44±0.18 vs. 0.96±0.07 mm, *P* = 0.041), suggesting that rostrocaudal migration is continuously progressing until 8 weeks post-transplantation. In contrast, when NSPCs were transplanted into the CA3 region of the right hippocampus of age-matched non-kindled rats, there was no apparent grafted cell migration from CA3 to the DG (Fig. S1). These results suggest that transplanted huNSPCs engraft robustly, migrate extensively, and survive for a long time after grafting into epileptic hippocampus. In the pilocarpine model, grafted cells also showed robust engraftment and long-term survival, and extensive distribution around the injection site when brains were analyzed 12 weeks following transplantation (Fig. S2).

### Differentiation of human NSPCs in epileptic rats following transplantation

**Figure 5:**
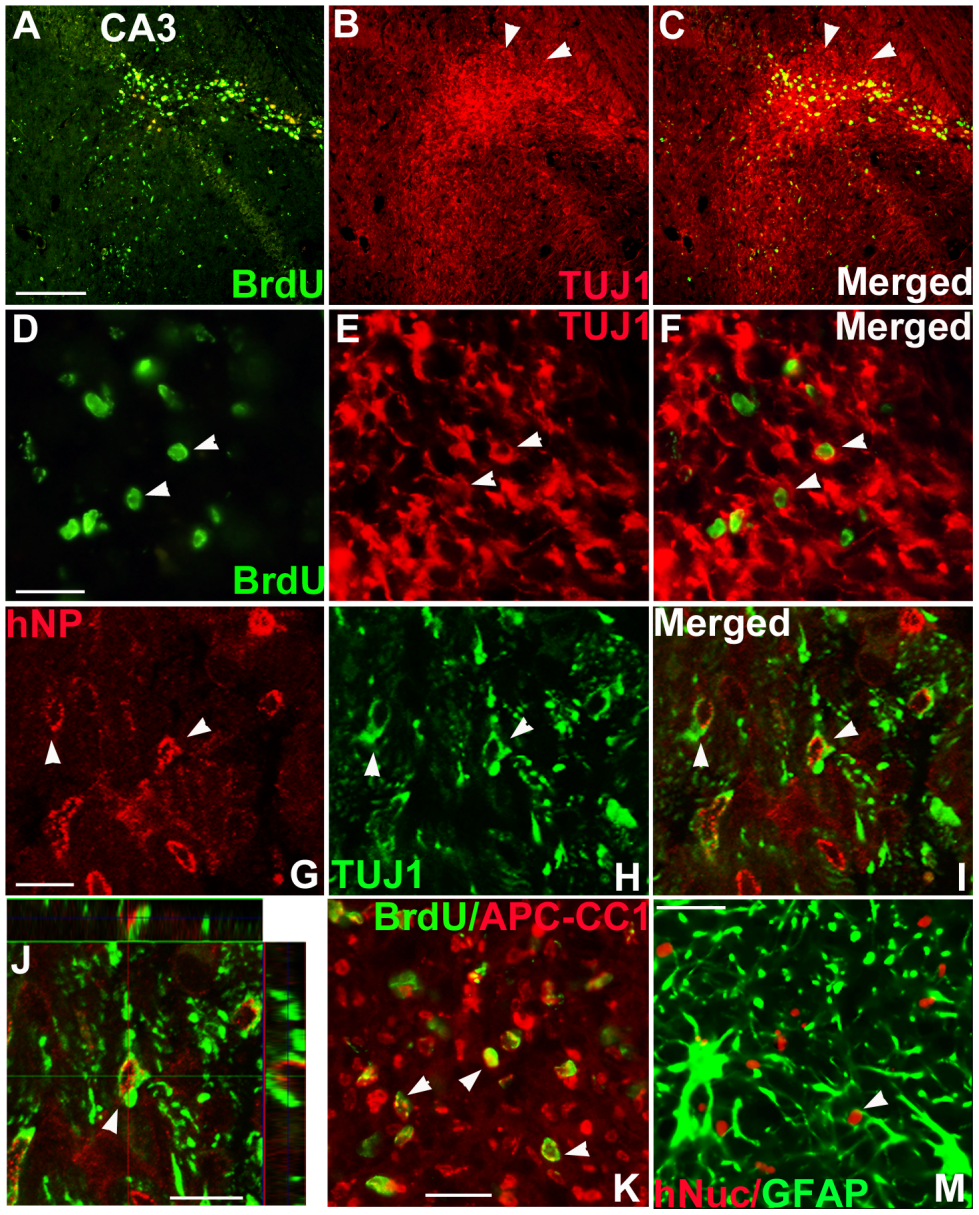
Differentiation of human NSPCs following transplantation into the hippocampus of kindled rats. (A–F) BrdU^+^ grafted cells (green) were co-stained with TUJ1 (red, arrowheads in B, C, E, F) in the CA3 region of the hippocampus (A–C) and fimbria (D–F) in rats. (G–J) hNP^+^ grafted cells (red, arrowheads in G, I, J) were co-localized with TUJ1 (green, arrowheads in H–J) in the hilus of the hippocampus. (J) Orthogonal view from confocal *z*-series showed that hNP (red) in nuclei and TUJ1 (green) in cytoplasm were expressed in the same cell. (K, M) Under the dual-filter microscope, BrdU^+^ grafted cells co-expressed APC-CC1 in the fimbria (arrows in K), and hNuc^+^ grafted cells co-expressed GFAP in the CA3 region (an arrow in M). Scale bar; 200 µm (A), 20 µm (D), 10 µm (G, J), 20 µm (K).

**Figure 6:**
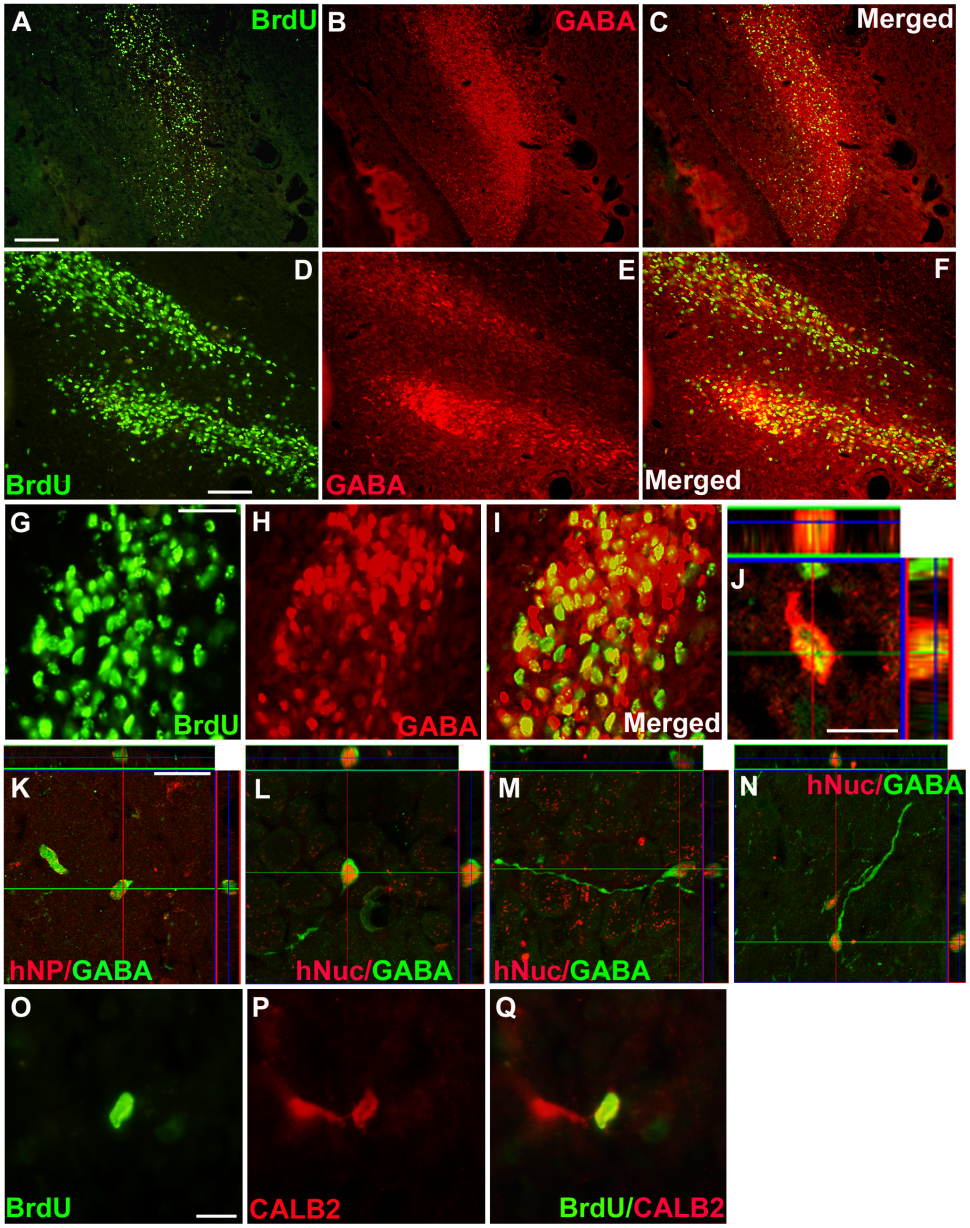
Differentiation of human NSPCs into GABAergic neurons in the hippocampus of kindled rats. (A–J) BrdU^+^ grafted cells (green, A, C, D, F, G, I, J), were co-labeled with GABA (red, B, C, E, F, H–J), in the molecular and granular layer of the dentate gyrus, hilus of the hippocampus (A–C), and the radiatum layer of the CA3 region (D–J) of rats. (G–I) A large fraction of BrdU^+^ grafted cells co-expressed GABA, as viewed under the dual-filter microscope. (J) Orthogonal view from confocal *z*-series visualized co-expression of BrdU (green) and GABA (red) in a grafted cell. (K–N) Orthogonal images show that hNP or hNuc^+^ grafted cells (red) co-expressed GABA (green) in different layers of the CA3: the stratum lucidum (K), stratum pyramidale (L and M), and stratum oriens (N). (O–Q) A BrdU^+^ grafted cells (green) were also co-labeled with calbindin2 (CALB2, red) in the stratum oriens around the CA3 area. Scale bar, 200 µm (A), 100 µm (D), 50 µm (G), 10 µm (J), 20 µm (K), 10 µm (O).

To study differentiation patterns of donor-derived cells, the whole hippocampal area of kindled rats was analyzed at 8 weeks following transplantation. BrdU^+^ cells were co-labeled with the early neuronal marker TUJ1 in the CA3 region of the hippocampus (Fig. 5A–C) and fimbria (Fig. 5D–F). Confocal microscopic images of hNP^+^-grafted cells demonstrated dual labeling with the TUJ1 antibody (Fig. 5G–J). Quantification revealed that donor-derived cells differentiated into TUJ1^+^ neurons (33.8±4.0%, *n* = 4), APC-CC1-expressing oligodendrocytes (27.7±6.2%, *n* = 4), and GFAP-expressing astrocytes (8.2±1.8%, *n* = 4) in the hippocampus (Fig. 5K, M). About 40% of GFAP-expressing astrocytes expressed GDNF (Fig. S3). Additionally, ∼24% of BrdU^+^ cells expressed GABA in the hippocampus and surrounding structures (*n* = 4; Fig. 6A–I). Confocal microscopy images showed that BrdU^+^ cells co-localized with GABA^+^ cells (Fig. 6J), and hNP^+^ cells dual-labeled with GABA had small bipolar neuronal processes (Fig. 6K–N). Most grafted cells, however, appeared not to show the morphological features of mature interneurons resembling host inhibitiory hippocampal interneurons. A few BrdU^+^ cells (∼3%) were found to express CALB2, a GABAergic interneuron subtype (Fig. 6O–Q). In the pilocarpine model, grafted NSPCs also differentiated into TUJ1^+^ neurons, OLIG2^+^ oligodendrocyte progenitors, and GFAP^+^ astrocytes, although a large number of cells expressed an undifferentiated cell marker, nestin (*n* = 4). Moreover, ∼21% of donor-derived cells expressed GABA and few of them expressed GDNF (Fig. S4). Most grafted cells also appeared not to show the morphological features of mature interneurons. These findings suggest that huNSPCs can give rise to neurons including GABAeric neurons albeit still immature, oligodendrocytes, and astrocytes in the epileptic hippocampus and adjacent structures in TLE models.

### Effect of human NSPC transplantation on kindled seizures and SRMS

**Figure 7:**
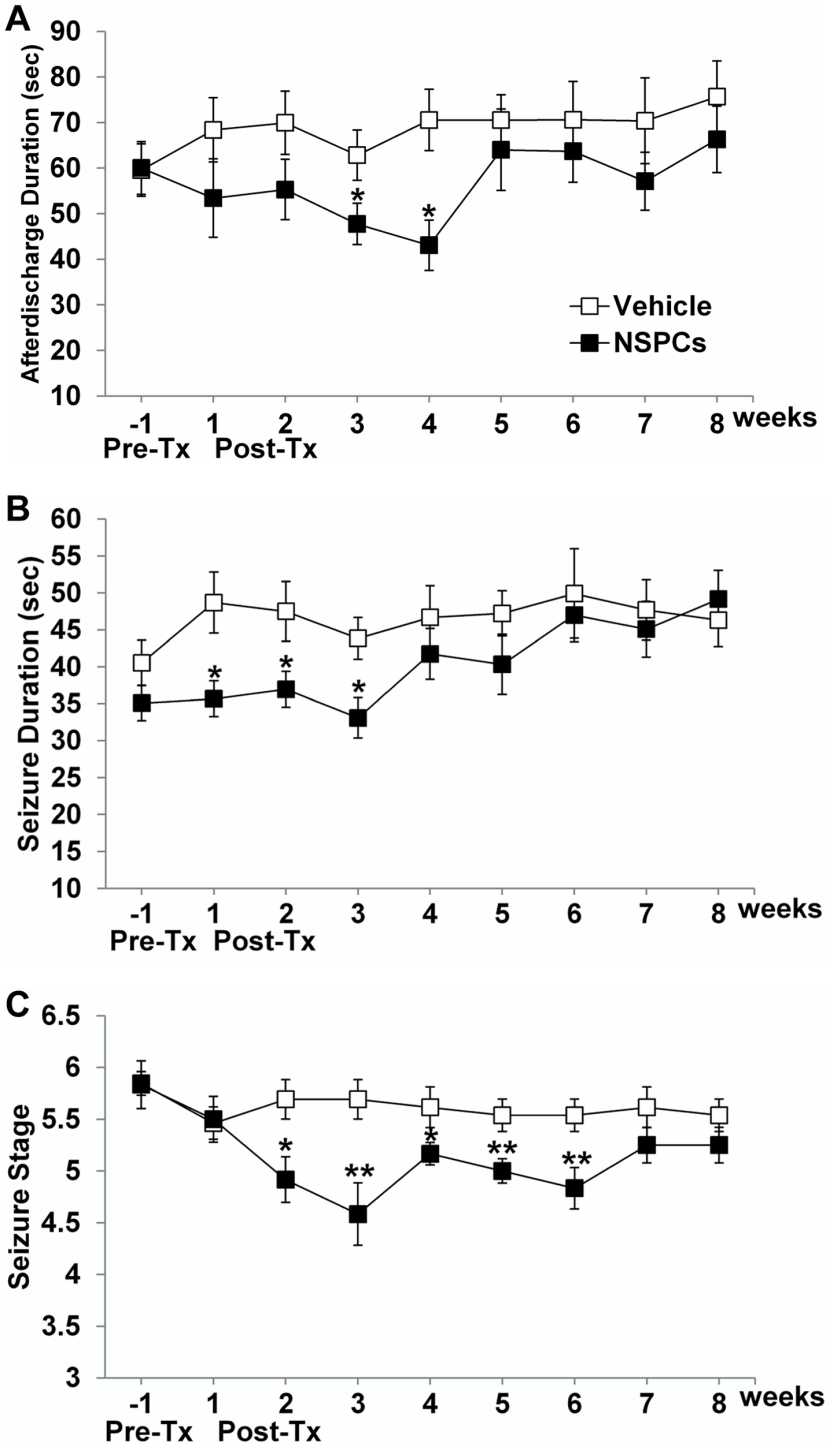
Effect of human NSPC grafting on hippocampal-kindled seizures in rats. The mean values of afterdischarge duration (ADD) in electroencephalograms (A), behavioral seizure duration (B), and seizure stage (C) between vehicle-injected and NSPC-transplanted groups were compared before (pre-Tx) and after NSPC grafting (post-Tx). Error bars indicate ±SEM. huNSPC grafting significantly reduced all three seizure parameters—ADD, behavioral seizure duration, and seizure stage—although this seizure-suppressing effect was not permanent. * Significantly different from the vehicle-injected group at *P*<0.05; ** significantly different from the vehicle-injected group at *P*<0.01.

The effect of huNSPCs transplantation on kindled seizure activity was evaluated weekly for 8 weeks following transplantation. Compared with the vehicle-injected group (*n* = 13), the transplanted group (*n* = 12) showed substantial decreases in three seizure parameters—ADD on EEG examination, behavioral seizure duration, and seizure stage—although no significant difference was observed in seizure parameters between pre-grafts in both groups (Fig. 7A–C). In the transplanted group, ADD decreased steadily until 4 weeks after grafting and significantly decreased compared with the vehicle group at the third week (24% reduction, *P* = 0.047) and fourth week (39% reductions, *P* = 0.005) (Fig. 7A).

Seizure duration declined significantly during the first, second, and third weeks after grafting (27%, 22%, and 25% reductions, respectively; *P*<0.05; Fig. 7B). Seizure stage also displayed a significant decrease from the second to sixth week (*P*<0.05; Fig. 7C). Furthermore, seizure parameters in NSPC-transplanted rats did not exceed those of vehicle-injected rats at almost every time point. Thus, huNSPCs grafting resulted in significant reductions in all seizure parameters in the kindling model. However, the significant anticonvulsant effect was not permanent, but disappeared slowly by the seventh week following transplantation (Fig. 7).

**Figure 8:**
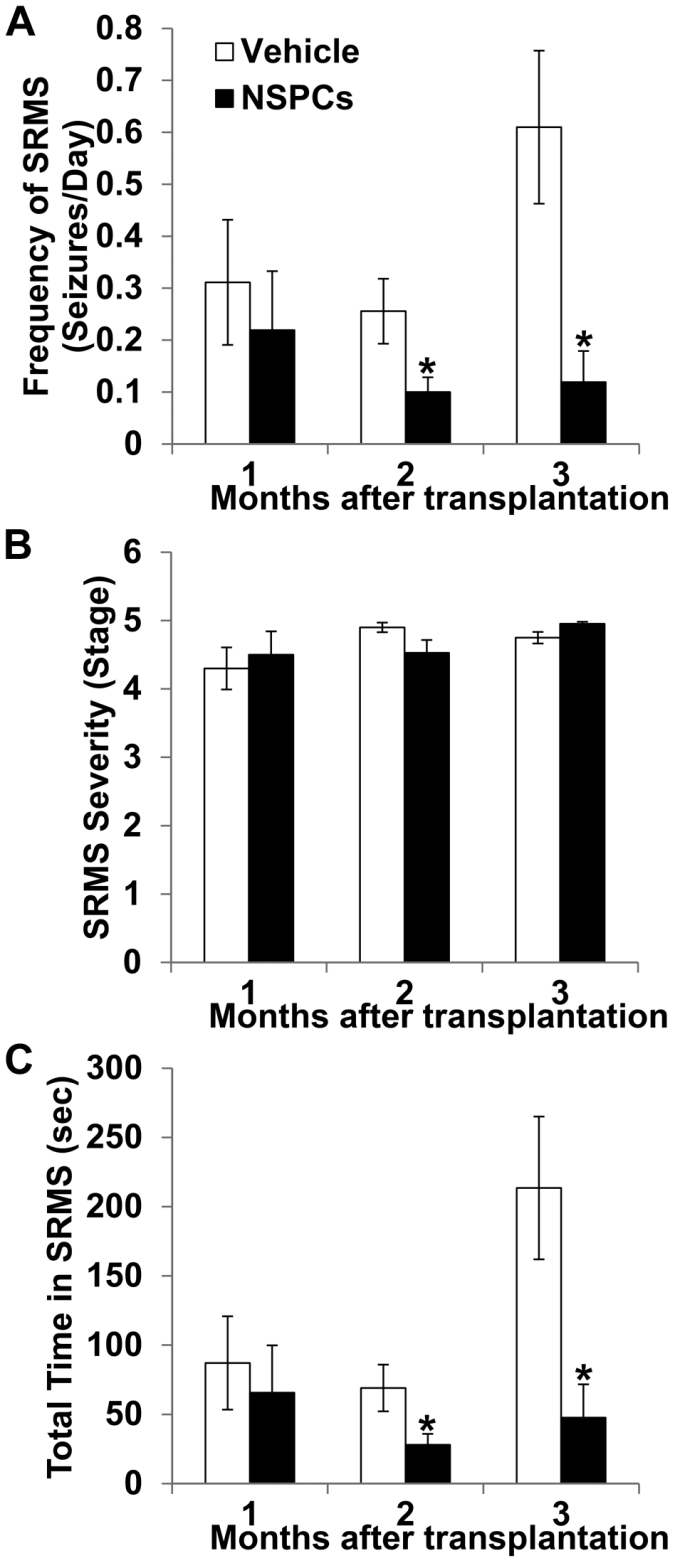
Effect of human NSPC grafting on spontaneous recurrent motor seizures (SRMSs) in pilocarpine-treated rats. The mean seizure frequencies (A), seizure stages (B) and total time spent in seizures (C) were calculated during 1, 2, and 3 months following transplantation in vehicle-injected and NSPC-transplanted groups. * Significantly different from the vehicle-injected group at *P*<0.05; error bars indicate ±SEM.

To examine the effect of huNSPCs transplantation on spontaneous seizures, we performed video monitoring in the pilocarpine model. Rats were video-monitored to document the emergence of SRMSs for 14–20 d after pilocarpine-induced SE. Previously, adult rats that underwent pilocarpine-induced SE for 1 h were reported to exhibit spontaneous seizures before 20 days after SE [24]. We also observed the emergence of at least one spontaneous seizure in all experimental animals. After confirmation of the occurrence of SRMSs, we injected NSPCs into both right and left hippocampus at 3 weeks after SE, and the rats were then video-monitored from 2 weeks to 3 months following transplantation. During the video monitoring period, we measured the frequency and severity of SRMS, and the total time spent in SRMS (Fig. 8). The mean frequencies of SRMSs in transplanted group (0.10±0.03 and 0.12±0.06 seizures/day at 2 and 3 months after grafting, respectively; *n* = 21) were significantly reduced compared with those in the vehicle group (0.26±0.06 and 0.61±0.15, respectively; *n* = 18; Fig. 8A). Thus, epileptic rats that received NSPC grafts into the hippocampus had significantly lower seizure frequencies as compared to vehicle-injected epileptic rats at 2 months (62% reduction, *P* = 0.029) and 3 months (80% reduction, *P* = 0.004) following transplantation. Seizure severity was usually stage 4 or 5. The mean seizure stage of SRMS was not different between both transplanted and vehicle groups (*P*>0.05; Fig. 8B). There were significant decreases in the average total time spent in SRMS at 2 and 3 months after grafting in the transplanted group (28.0±8.0 and 47.6±24.0 s, respectively) as compared to the corresponding time points in the vehicle group (69.0±16.9 and 213.5±51.5 s, respectively) (*P* = 0.033 and *P* = 0.007, respectively; Fig. 8C). The mean duration of individual SRMSs was not different in the transplanted group than in the vehicle group at any time point (*P*>0.05). The data suggest that huNSPCs grafting resulted in long-term significant attenuation of SRMS in a representative TLE model.

### Effect of human NSPC transplantation on learning and memory function

**Figure 9:**
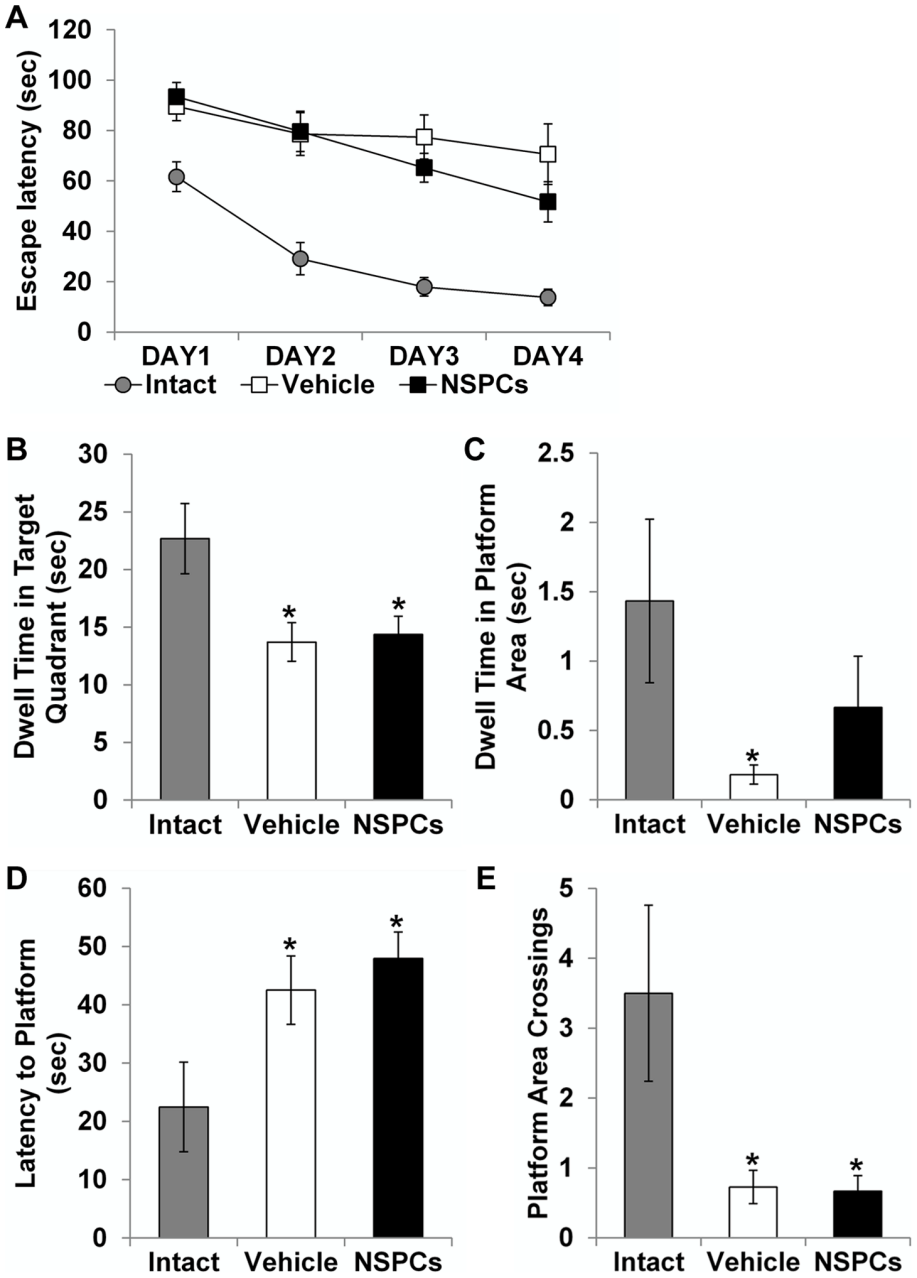
Effect of human NSPC grafting on spatial learning and memory function in the pilocarpine-treated rats. (A) Latency to locate the hidden platform (escape latency) was recorded. In the age-matched intact controls, escape latency decreased gradually and significantly over the 4 days of testing, indicating excellent spatial learning. In contrast, NSPC- and vehicle-injected epileptic rats showed markedly longer latency to escape the maze than intact controls (*P* = 0.024 and *P* = 0.006, respectively). (B–E) During probe testing on day 5, parameters of memory retention were measured in the three groups: dwell time in the target quadrant (B), dwell time in the platform area (C), latency to the platform (D), and platform area crossings (E). Vehicle-injected epileptic rats exhibited significant deficits in memory retention in terms of all parameters. NSPC-transplanted epileptic rats also showed significant memory deficits, except dwell time in the platform area. * Significantly different from age-matched intact controls at *P*<0.05; error bars indicate ±SEM.

Epilepsy in patients is often accompanied by cognitive decline [46]. Thus, we examined whether huNSPC transplantation could affect performance in a Morris water maze test, which assesses hippocampal-dependent learning and memory function. To estimate the effect of NSPC transplantation on learning and memory function in the pilocarpine model, we performed water maze testing at 3 months post-grafting. Age-matched intact controls (*n* = 6) showed the marked decreases in the escape latencies over the sessions, indicating normal spatial learning ability. However, epileptic rats in the vehicle group (*n* = 11) did not improve significantly in locating the submerged platform over the trials (Fig. 9A), consistent with other reports, demonstrating impaired spatial learning [47], [48]. During the probe test to evaluate the memory function, epileptic rats in the vehicle group spent significantly shorter times in the target quadrant and in the platform area (Fig. 9B, C), took longer to reach the platform area (Fig. 9D), and barely crossed the platform area compared with intact controls (Fig. 9E). In the transplantation group (*n* = 12), rats did not show amelioration in overall spatial learning or memory function, and were not considerably different from the vehicle-injected group.

We performed water maze testing at 9 weeks post-grafting in fully kindled rats. Kindled rats of the vehicle-injected and NSPC-transplanted groups (*n* = 11 and 8, respectively) showed gradual decline in escape latency across training days. The probe test was performed at 24 h after the last training day to evaluate memory function. NSPC-grafted group were indistinguishable in all parameters of reference memory (latency to reach the platform area, platform area crossings, dwell time in the target quadrant, and dwell time in the platform area) compared to vehicle-injected group. No significant difference was observed in the learning and memory function between both groups (data not shown). Thus, huNSPCs transplantation did not interfere with the ability of spatial learning and memory retention in kindled rats.

### Effect of human NSPC grafting on the expression of GDNF in host hippocampal astrocytes

**Figure 10:**
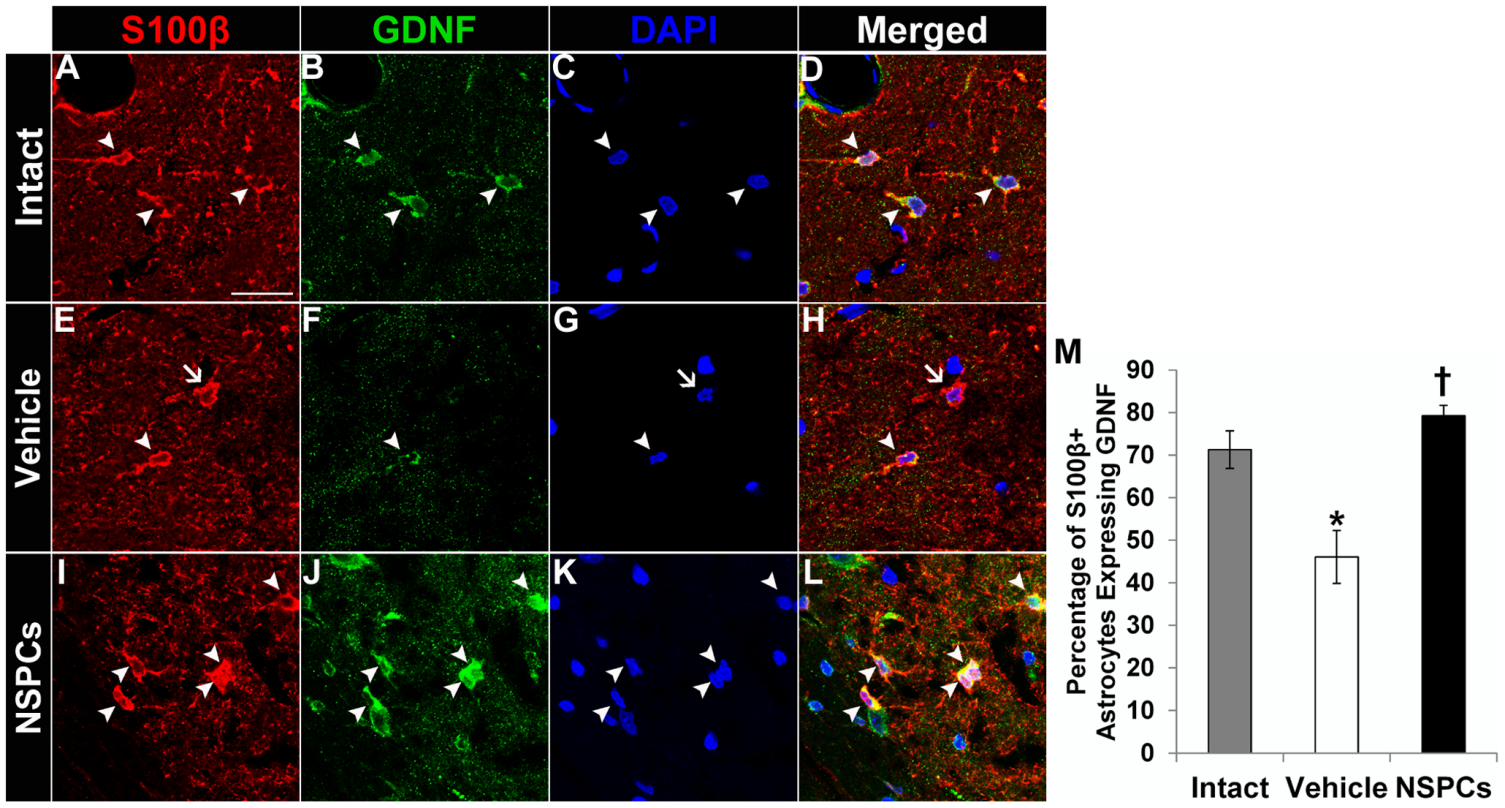
Effect of human NSPC grafting on the expression of GDNF in host hippocampal astrocytes. GDNF expression in S100β^+^ hippocampal astrocyte was observed in an age-matched intact control (A–D), vehicle-injected pilocarpine-treated (E–H), and NSPC-transplanted pilocarpine-treated rats (I–L). Nuclei were counterstained with DAPI (C, G, and K). Arrowheads in A–L indicated S100β/GDNF double-labeled cells. Arrows in E, G and H denoted S100β^+^ host hippocampal astrocytes that were devoid of GDNF immunoreactivity in vehicle-injected epileptic rats. Scale bar, 50 µm. (M) The bar chart represents percentages of S100β^+^ astrocytes expressing GDNF in the CA3 region of the hippocampus in the three groups. There was a significant difference between intact controls and vehicle-injected epileptic rats (*P* = 0.022) and between vehicle-injected and NSPC-transplanted epileptic rats (*P* = 0.038). * Significantly different from the age-matched intact control group at *P*<0.05; † significantly different from vehicle-injected group at *P*<0.05; error bars indicate ±SEM.

Increased GDNF levels in hippocampal astrocytes of the epileptic brain are known to suppress seizures [49], [50]. In this study, few huNSPC-derived cells after grafting differentiated into GDNF-expressing astrocytes in the hippocampus in either TLE model. However, NSPC transplantation induced GDNF expression in a large part (>80%, *n* = 6) of host hippocampal astrocytes in the pilocarpine-treated TLE model, whereas the level of GDNF expression of host astrocytes was ∼72% (*n* = 4) in age-matched intact controls and ∼46% (*n* = 6) in vehicle-injected epileptic rats (Fig. 10). This suggested that the level of GDNF expression was restored to closer to that of the intact controls after NSPCs grafting. Thus, the induction of GDNF expression in host hippocampal astrocytes by huNSPCs transplantation may be involved in suppressing seizures.

### Effect of human NSPC transplantation on aberrant mossy fiber sprouting

**Figure 11:**
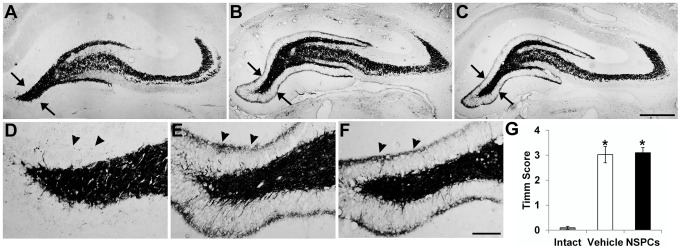
Effect of human NSPC grafting on aberrant mossy fiber sprouting in pilocarpine-treated animals. (A–F) Timm staining exhibits mossy fiber staining from age-matched intact control (A, D), vehicle-injected pilocarpine-treated (B, E) and huNSPCs-transplanted pilocarpine-treated rats (C, F) 3 months following transplantation. D–F are high-magnification views of areas indicated by arrows in A–C, respectively. Arrowheads in D–F indicate the supragranular region of the dentate gyrus. Pilocarpine-treated rats reveal mossy fiber sprouting with an increased density of Timm granules in the supragranular region (B, C, E, F), whereas control rats did not (A, D). Scale bar, 500 µm (C), 100 µm (F). (G) NSPC-transplanted and vehicle injected rats had higher Timm scores than the control group (*P<0.05). Error bars, SEM. No significant difference regarding Timm score was found between NSPC- and vehicle-injected groups.

Aberrant sprouting of mossy fibers into the inner molecular layer of the DG of the hippocampus is one of the best-known structural changes in TLE models [2]–[5]. Because mossy fiber sprouting (MFS) is known to be linked to increased seizure susceptibility in TLE [51], we examined whether huNSPCs grafting could reduce MFS in the pilocarpine model. To visualize mossy fibers, we performed Timm staining that selectively labeled zinc-containing mossy fibers and recorded the Timm score to evaluate the extent of MFS. In age-matched intact rats (*n* = 4), Timm staining was nearly absent in the supragranular region of the DG (Fig. 11A, D). However, compared with intact rats, prominent Timm granules were present in the supragranular region in pilocarpine-treated rats, indicating aberrant MFS (Fig. 11B, C, E, F). The statistical analysis showed that the Timm score for MFS was not significantly different between vehicle-injected and NSPC-transplanted rats (*n* = 4 and 4, respectively; *P* = 1.0; Fig. 11G). This finding indicates that the significant reduction in seizure frequency seen with huNSPCs transplantation was not the result of a change of aberrant mossy fiber sprouting in the dentate gyrus.

## Discussion

This study presents the first evidence that human fetal telencephalon-derived NSPC transplantation into the adult epileptic rat brain exerts a therapeutic effect in suppressing kindling-induced evoked seizures and spontaneous recurrent seizures in the pilocarpine-induced TLE model. In the kindling model, huNSPCs grafting showed a considerable reduction in ADD, seizure duration, and seizure stage for several weeks following transplantation. However, the seizure-restraining effect was not lasting, but slowly disappeared by ∼6 weeks after grafting, although the grafts survived and contained GABA-expressing cells over a longer video-EEG monitoring period (>8 weeks).

While the kindling model exhibits some of the prominent features of TLE, the disadvantages of kindling are the deficiency of spontaneous seizures and physiological and morphological similarities of hippocampal lesions compared with human mesial TLE. In contrast, the pilocarpine model displays characteristic features of TLE—spontaneous seizures acquired after a brain insult, loss of cognitive function, behavioral alterations, and poor responses to AEDs—which mimic human TLE in adults [2], [5], [24], [52]. Thus, we then transplanted huNSPCs into the bilateral hippocampus of pilocarpine-induced epileptic rats after SRMSs emerged.

Epileptic animals showed a marked decrease in seizure frequency and total time spent in seizure at 2 and 3 months after cell grafting. Furthermore, grafted cells not only showed extensive migration, robust engraftment, long-term survival, and differentiation into three CNS neural cell types around the injection site, but also caused the addition of a great number of GABAergic interneurons into the hippocampus in both kindling and pilocarpine-treated TLE models. However, although huNSPCs grafting into the adult epileptic brain exerted a considerable anticonvulsant effect, the decline in cognitive function commonly associated with epilepsy did not improve.

Loss of inhibitory interneurons is seen commonly in the hippocampus of TLE animal models and patients with epilepsy [5], [53]-[55]. To examine seizure suppression by specifically increasing the inhibitory tone, fetal hippocampal precursors or cells genetically engineered to express GABA were transplanted into animal models [9], [29], [56], [57]. These studies reported modest or transient antiseizure effects, suggesting that cell-based therapies that augment inhibitory neural transmission could be therapeutic in patients with TLE.

Recent works have focused on fetal MGE precursor cells as donor cell types for treating TLE because MGE is the origin of most cortical, hippocampal, and striatal GABAergic interneurons in the developing brain [43], [58], [59]. Transplanted fetal MGE precursors showed widespread migration and inhibitory synapse formation onto host cortical pyramidal neurosns in intact brain [31], [60], and reduced the number of electrographic seizures in mice with a potassium channel mutation [18]. When freshly harvested mice fetal MGE precursors were grafted into the hippocampus of adult mice with pilocarpine-treated TLE, the incidence of seizures markedly decreased and behavioral deficits improved [19]. Almost all grafted MGE cells exhibited not only morphologically mature inhibitory interneurons, and expressed typical molecules of interneurons, but also differentiated into electrophysiologically functional inhibitory neurons and integrated into the local circuitry. These results suggest that fetal MGE precursor grafts appear to be a strong tool to suppress seizures and recover behavioral deficits in severe adult epileptic mice. However, despite these promising findings, obtaining freshly dissociated human fetal MGE precursors for TLE therapy is impractical. Thus, abundant generation of comparable and safe MGE precursors from human ES or iPSCs will be necessary before therapeutic application. Recently, the technology for producing MGE-like precursors from human ES or iPSCs is currently being developed [21], [26], [61].

Another recent study showed that transplantation of rat fetal MGE-derived NSCs, which were expanded in culture as neurospheres, into adult rats with chronic TLE suppressed SRMS efficaciously [17]. In that study, adequate numbers of donor cells for grafting in epilepsy were obtained by expanding MGE-derived NSCs in dishes as neurospheres. Transplanted cells differentiated into significant numbers of GABAergic interneurons and anticonvulsant neurotrophic factor GDNF-expressing astrocytes [49], which produced a sustained antiseizure effect over a prolonged period of time in a chronic TLE model. However, prior to clinical application of MGE-derived NSCs, developing additional cell grafting methods will be necessary for enhancing the production of graft-derived GABAergic neurons, the level of overall seizure suppression, and the cognitive performance.

Although mouse fetal MGE precursors and rat MGE-derived NSCs grafts markedly attenuate seizures in epileptic brains of adult animals, the production of an equivalent MGE-like human stem cells, such as those derived from developing or adult brain tissues, or from ES or iPSCs, will be required before any application to the clinic.

In this study, we prepared huNSPCs derived from the telencephalic brain tissue of an aborted fetus at 13 weeks of gestation and maintained them in serum-free growth medium by passaging through the dissociation of bulk neurospheres in a Good Manufacturing Practice facility. Under these culture conditions, the doubling time of huNSPCs was 4–5 days, and cells proliferated continuously and generated a great number of progeny for over 1 year. The vast majority of cells (>99%) within the neurospheres expressed immature neural stem cell or progenitor markers: nestin, vimentin, GFAP, Pax6, and Sox2 [22]. On cytogenetic analysis, huNSPCs were diploid and retained a normal karyotype after long-term passage, and an array-based comparative genomic hybridization (aCGH) study also showed no genomic alteration (Fig. S5).

Under differentiation conditions, huNSPCs could differentiate not only into multiple neural cell lineages but also into interneuron populations, although some of them have generated neurons of diverse neurotransmitter phenotypes [22]. In this study, huNSPCs were not precisely derived from the GE of an aborted fetal brain, but broadly from the dorsal and ventral part of the telencephalon. However, NSPCs expressed *NKX2.1* transcription factor, which is required for specifying MGE-derived GABAergic interneurons [26], [42], and abundantly *NR2F2*, which is preferentially expressed in the CGE [26], [44], [45]. These data exhibit that NSPCs contain a substantial fraction of GE-derived stem cells which were recently reported to be the primary sources of cortical interneurons in human [62]. Additionally, NSPCs expressed the vental telencephalic GABAergic neuronal lineage markers (*ASCL1* and *DLX2*), GABAergic neuronal markers (*GAD1*, *SLC32A1*, and *SLC6A1*), and interneuron subtype markers (*NPY*, *SST*, and *CALB2*). Moreover, ∼26% of NSPC-derived differentiated cells expressed GABA, ∼11% of the cells expressed GABA-synthesizing enzyme GAD2, and the cells released GABA into the culture medium in response to depolarization due to high potassium.

Thus, huNSPCs, derived from a single donated fetal brain, could be expanded in culture for extended periods and cryopreserved into cell banks, from which adequate amounts of cells could be prepared for transplantation into patients with epilepsy. Additionally, huNSPCs could give rise to a considerable fraction of GABAergic interneurons after grafting into the hippocampus of patients with TLE.

In this study, significant repression of SRMSs by huNSPCs grafts appeared to be caused by the addition of GABAergic neurons albeit still immature. Regarding GABAergic neurons, huNSPCs transplantation additionally provided ∼28,000 GABAergic neurons into the hippocampus in the kindling model and ∼24,000 GABAergic neurons into each hippocampus in the pilocarpine-treated model. This addition is substantial, considering that GABAergic function decreases in TLE [5], [53]–[55], [63]–[65] and grafted cells release GABA, which facilitates the antiseizure effect.

Although huNSPCs grafting resulted in significant reductions in all seizure parameters in the kindling model, the significant seizure-suppressing effect was not permanent, but disappeared slowly by the seventh week following transplantation. Previous studies also reported that simply GABA-secreting cell grafts induced transient antiseizure effects [9], [29], [56], [57]. This transient antiseizure effect of cell grafting has been observed previously in most rodent studies [9], [66]. This may be not only a consequence of decreased implanted cell viability or poor integration into epileptic hippocampal circuits, but also of a decline in GABA release from grafted cells, desensitization of the GABA receptors [9], or relatively low number of grafted cells-derived mature GABAergic interneurons.

In the pilocarpine-treated model, huNSPCs grafting showed a progressive reduction in seizure frequency and total time spent in seizure over the post-grafting survival period, and many donor-derived GABAergic neurons could be found at 3 months following transplantation. However, most grafted cells appeared not to show the morphological features of mature interneurons resembling host inhibitiory hippocampal interneurons. Thus, precise electrophysiological, morphological, and molecular studies are needed to observe some possibilities of functional synaptic integration of grafted GABAergic neurons on the host hippocampal circuitry.

A prior study has reported that rat fetal MGE-derived NSCs grafting into rats with chronic epilepsy restrained spontaneous seizures by the supply of new donor-derdived GDNF-positive cells with recovery of GDNF expression in host hippocampal astrocytes [17]. In this study, few huNSPC-derived cells after grafting differentiated into GDNF-expressing astrocytes in either TLE model. However, NSPC transplantation induced GDNF expression in host hippocampal astrocytes in the pilocarpine-treated TLE model. huNSPCs express FGF-2 at a high level and FGF-2 is known to induce GDNF expression in astrocytes [67], [68]. Increased GDNF levels in hippocampal astrocytes of the epileptic brain are known to suppress seizures [49], [50]. Thus, the induction of GDNF expression in host hippocampal astrocytes by huNSPCs transplantation may be involved in suppressing seizures.

huNSPCs grafting could not reverse spatial learning and memory function in the pilocarpine-treated TLE model. As described above, when a large part of transplanted fetal MGE precursor cells differentiated into mature inhibitory interneurons and integrated functionally into the existing hippocampal neuronal network in the TLE model, a marked reduction in seizures and some restoration of behavioral deficits, including spatial learning and memory function, could be observed [19]. Thus, to not only induce remarkable long-term seizure suppression but also rescue accompanying cognitive deficits in epileptic animals, new additional strategies of huNSPCs grafting should be developed to improve survival and neuronal differentiation of grafted cells, and the yield of graft-derived mature GABAergic interneurons, which functionally integrate into the epileptic hippocampal circuitry. Additionally, restoration of cognitive function may require grafting of NSPCs that are capable of secreting various therapeutic molecules, such as growth factors, antiepileptic peptides, neurogenesis-enhancing factors, and anti-inflammatory/immunomodulatory factors.

Taken together, our results provide the first evidence that human fetal brain-derived NSPC transplantation into the hippocampus has therapeutic potentials for managing TLE, particularly with regard to seizure suppression. huNSPCs, derived from a single donated fetal brain, could be expanded in culture for a long time, from which sufficient numbers of cells could be prepared for transplantation into patients with epilepsy. Transplanted human cells showed extensive migration, robust engraftment, long-term survival, differentiation into three CNS neural cell types, and a large number of GABAergic interneurons around the grafted sites. However, before any clinical application, further studies to both increase the yield of NSPC grafts-derived functionally integrated GABAergic neurons and improve cognitive deficits are still needed.

## Supporting Information

Figure S1 **Engraftment and distribution of human NSPCs following transplantation into the hippocampus of age-matched non-kindled rats.** (A) A schematic figure illustrates the distribution of grafted cells in intact non-kindled rats 8 weeks after transplantation into the CA3 region of the right hippocampus. Grafted cells were mostly placed around the injection site (arrow), and seldom observed in the dentate gyrus (DG) and hilus (HI) of the hippocampus. (B) SC121^+^ grafted cells—visualized using fluorescein—were shown around the injection site. Scale bar, 100 µm.
(TIF)

Figure S2 **Engraftment and distribution of human NSPCs following transplantation into the hippocampus of pilocarpine-treated rats.** hNuc^+^ cells—visualized using Texas Red—were located in in the radiatum layer of the CA1 and CA3 regions, lacunosum molecular layer of the CA1 region, molecular and granular layer of the dentate gyrus, and hilus of the hippocampus when brains were analyzed 3 months post-grafts. Dotted line denotes the boundary between hilus (h) and granular layer of the dentate gyrus (DG). Scale bar, 200 µm.
(TIF)

Figure S3 **Expression of GDNF from transplanted human NSPCs-derived astrocytes in the hippocampus of kindled rats.** (A–D) Anti-human specific GFAP SC123^+^ grafted cells, visualized with Texas Red (A) were co-localized with GDNF, identified using fluorescein (B). Nuclei were counterstained with DAPI (C). (E) Orthogonal view from confocal *z*-series showed that SC123 and GDNF were co-expressed in cytoplasm of the same cell. Scale bar; 20 µm (A, E). GDNF, glial-derived neurotrophic factor.
(TIF)

Figure S4 **Differentiation of human NSPCs following transplantation into the hippocampus of pilocarpine-treated rats.** (A–D) A large number of hNuc^+^ grafted cells expressed undifferentiated cell marker, nestin in the hippocampus of pilocarpine-treated rats. (E–H) About 10% of hNuc^+^ grafted cells differentiated into TUJ1^+^ neurons (arrowheads in F, G). (I–L) ∼60% of hNuc^+^ grafted cells expressed GFAP. (M–P) A few anti-human specific cytoplasm SC121^+^ grafted cells were co-localized with Olig2, oligodendrocyte progenitor marker (arrows in M–O). (Q–T) ∼21% of hNuc^+^ grafted cells were co-labeled with GABA. (D, H, L, P, T) Orthogonal view from confocal *z*-series visualized co-expression of grafted cells (red) and various cell markers (green) in the same cell. Scale bar; 50 µm (A), 20 µm (D).
(TIF)

Figure S5 **Cytogenetic analysis of human NSPCs.** (A) Karyotyping and G-banding analysis of huNSPCs revealed a normal diploid karyotype at passage number 27 (46, XY). (B) Array CGH analysis of huNSPCs further confirmed their normal karyotype.
(TIF)

Table S1 Primers used for quantitative real-time PCR.
(DOCX)
