# Supplementary figures and images for: Human Fetal Brain-Derived Neural Stem/Progenitor Cells Grafted into the Adult Epileptic Brain Restrain Seizures in Rat Models of Temporal Lobe Epilepsy
Source: PLoS One. 2014 Aug 8;9(8):e104092. doi: 10.1371/journal.pone.0104092 (PMC4126719; doi:10.1371/journal.pone.0104092)

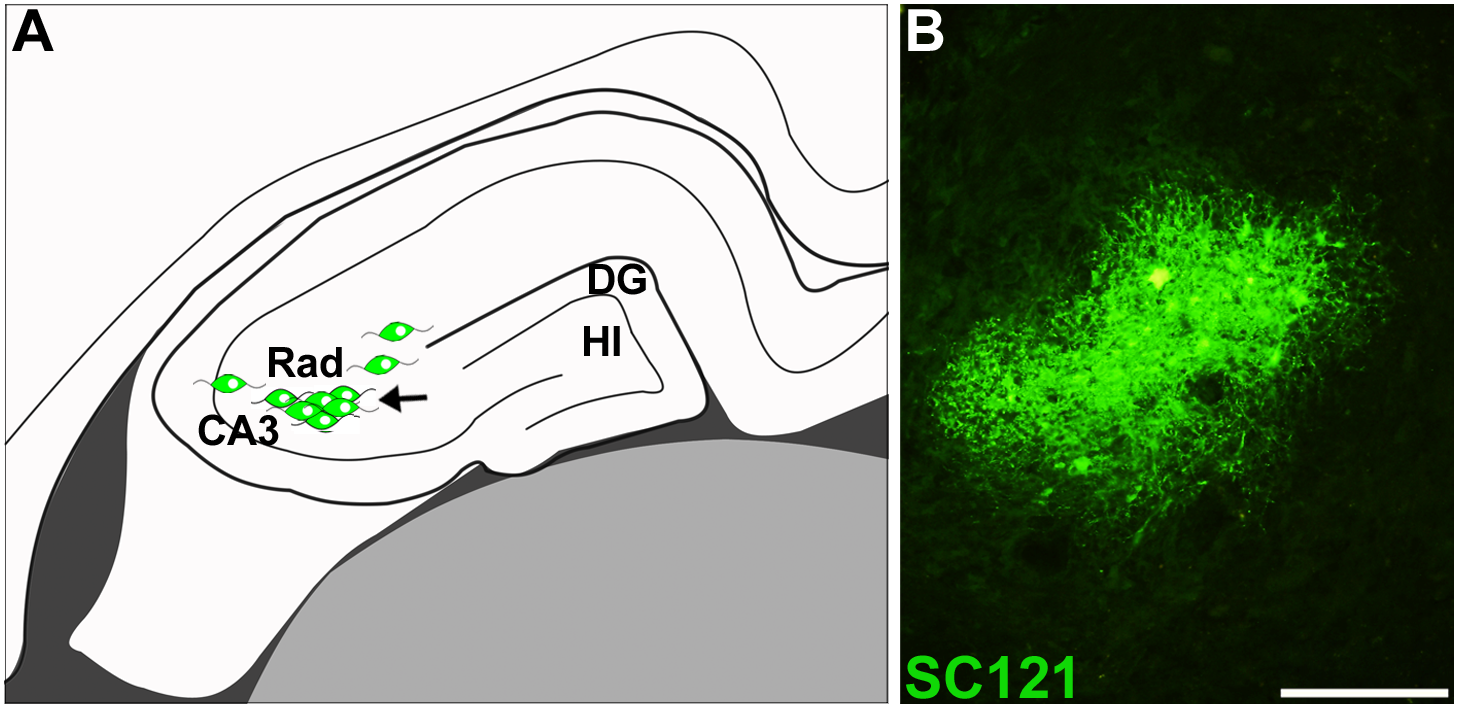

Supplement: Figure S1 — Engraftment and distribution of human NSPCs following transplantation into the hippocampus of age-matched non-kindled rats. (A) A schematic figure illustrates the distribution of grafted cells in intact non-kindled rats 8 weeks after transplantation into the CA3 region of the right hippocampus. Grafted cells were mostly placed around the injection site (arrow), and seldom observed in the dentate gyrus (DG) and hilus (HI) of the hippocampus. (B) SC121+ grafted cells—visualized using fluorescein—were shown around the injection site. Scale bar, 100 µm. (TIF) [file pone.0104092.s001.tif]

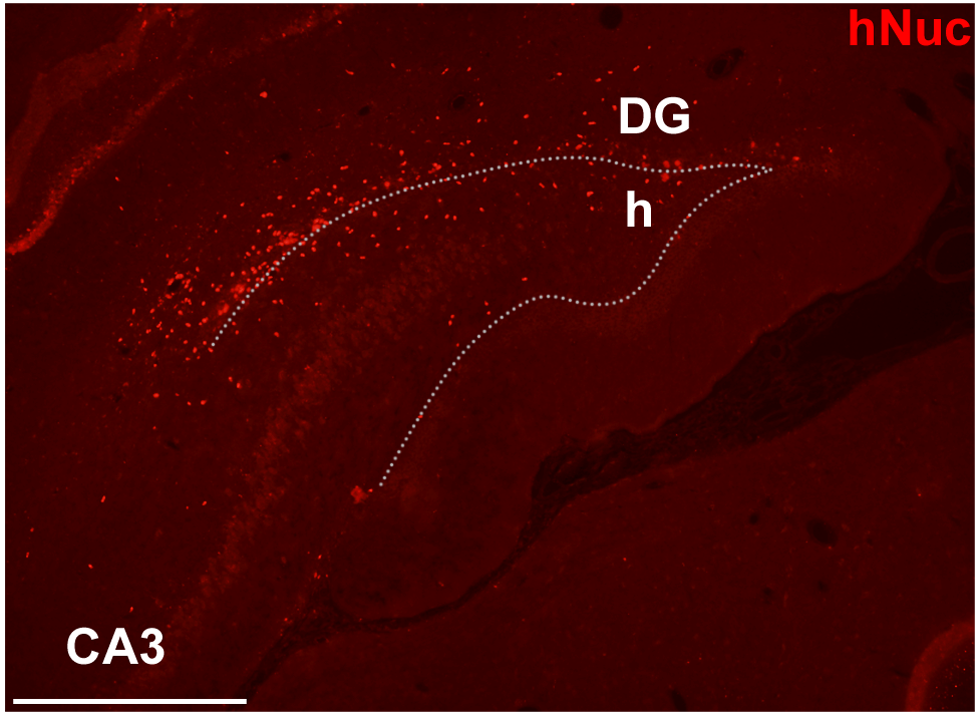

Supplement: Figure S2 — Engraftment and distribution of human NSPCs following transplantation into the hippocampus of pilocarpine-treated rats. hNuc+ cells—visualized using Texas Red—were located in in the radiatum layer of the CA1 and CA3 regions, lacunosum molecular layer of the CA1 region, molecular and granular layer of the dentate gyrus, and hilus of the hippocampus when brains were analyzed 3 months post-grafts. Dotted line denotes the boundary between hilus (h) and granular layer of the dentate gyrus (DG). Scale bar, 200 µm. (TIF) [file pone.0104092.s002.tif]

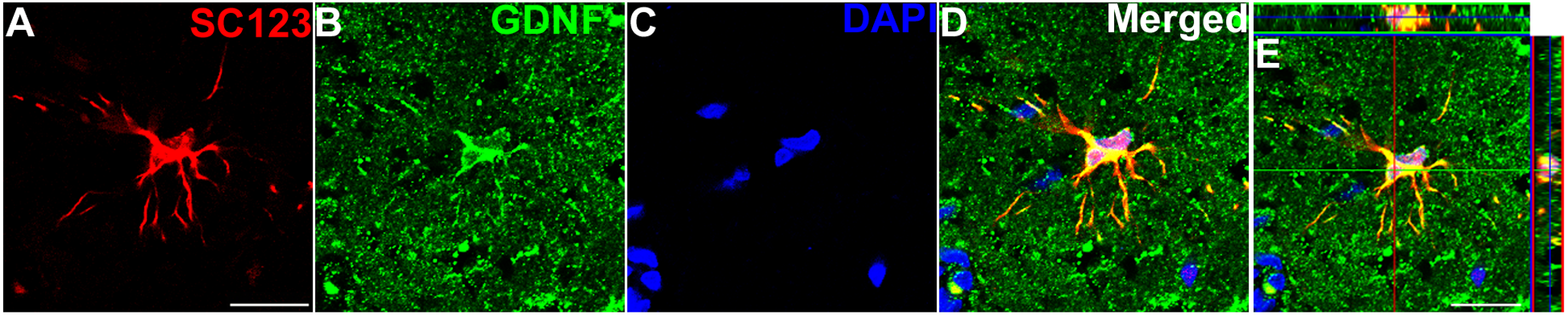

Supplement: Figure S3 — Expression of GDNF from transplanted human NSPCs-derived astrocytes in the hippocampus of kindled rats. (A–D) Anti-human specific GFAP SC123+ grafted cells, visualized with Texas Red (A) were co-localized with GDNF, identified using fluorescein (B). Nuclei were counterstained with DAPI (C). (E) Orthogonal view from confocal z-series showed that SC123 and GDNF were co-expressed in cytoplasm of the same cell. Scale bar; 20 µm (A, E). GDNF, glial-derived neurotrophic factor. (TIF) [file pone.0104092.s003.tif]

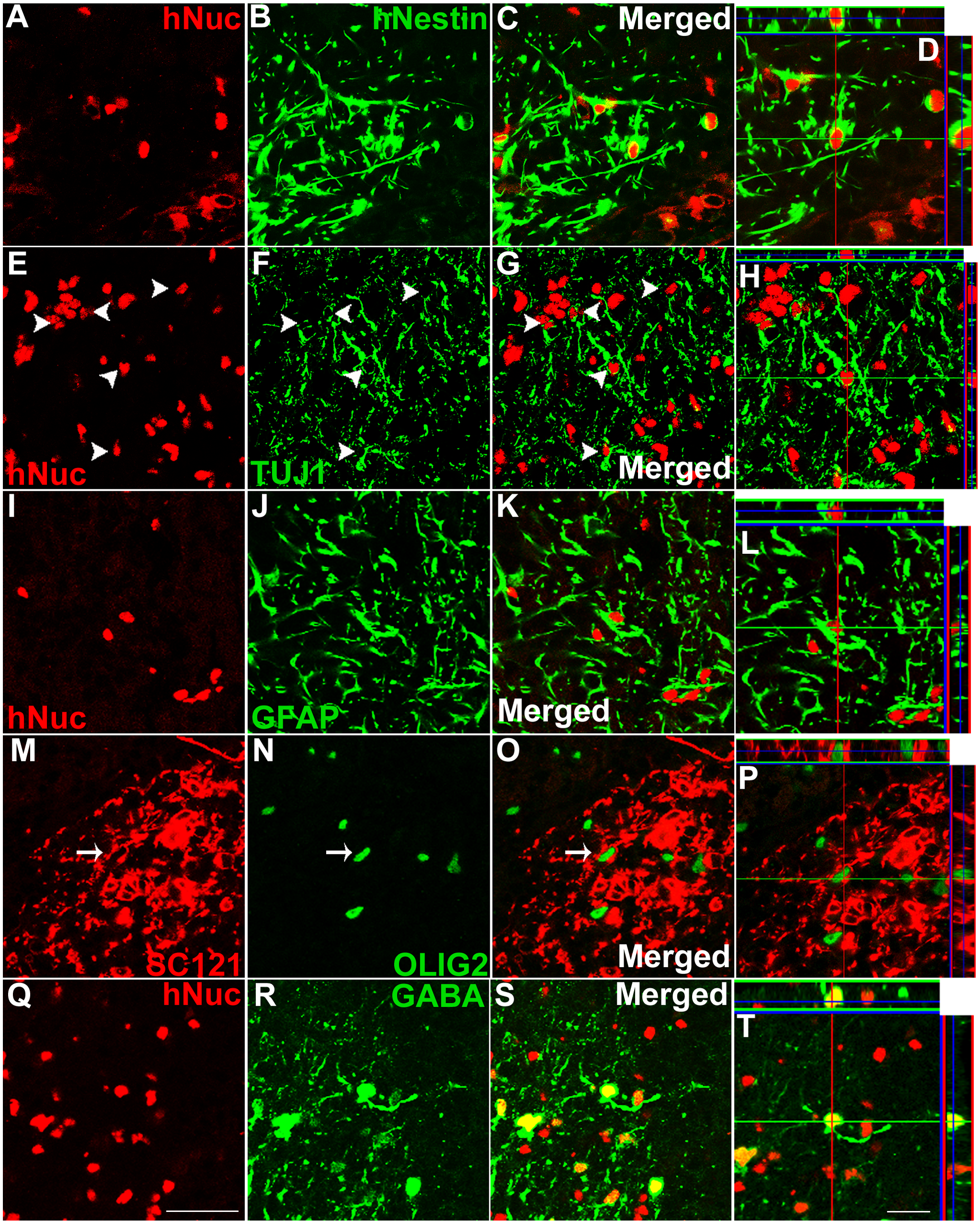

Supplement: Figure S4 — Differentiation of human NSPCs following transplantation into the hippocampus of pilocarpine-treated rats. (A–D) A large number of hNuc+ grafted cells expressed undifferentiated cell marker, nestin in the hippocampus of pilocarpine-treated rats. (E–H) About 10% of hNuc+ grafted cells differentiated into TUJ1+ neurons (arrowheads in F, G). (I–L) ∼60% of hNuc+ grafted cells expressed GFAP. (M–P) A few anti-human specific cytoplasm SC121+ grafted cells were co-localized with Olig2, oligodendrocyte progenitor marker (arrows in M–O). (Q–T) ∼21% of hNuc+ grafted cells were co-labeled with GABA. (D, H, L, P, T) Orthogonal view from confocal z-series visualized co-expression of grafted cells (red) and various cell markers (green) in the same cell. Scale bar; 50 µm (A), 20 µm (D). (TIF) [file pone.0104092.s004.tif]

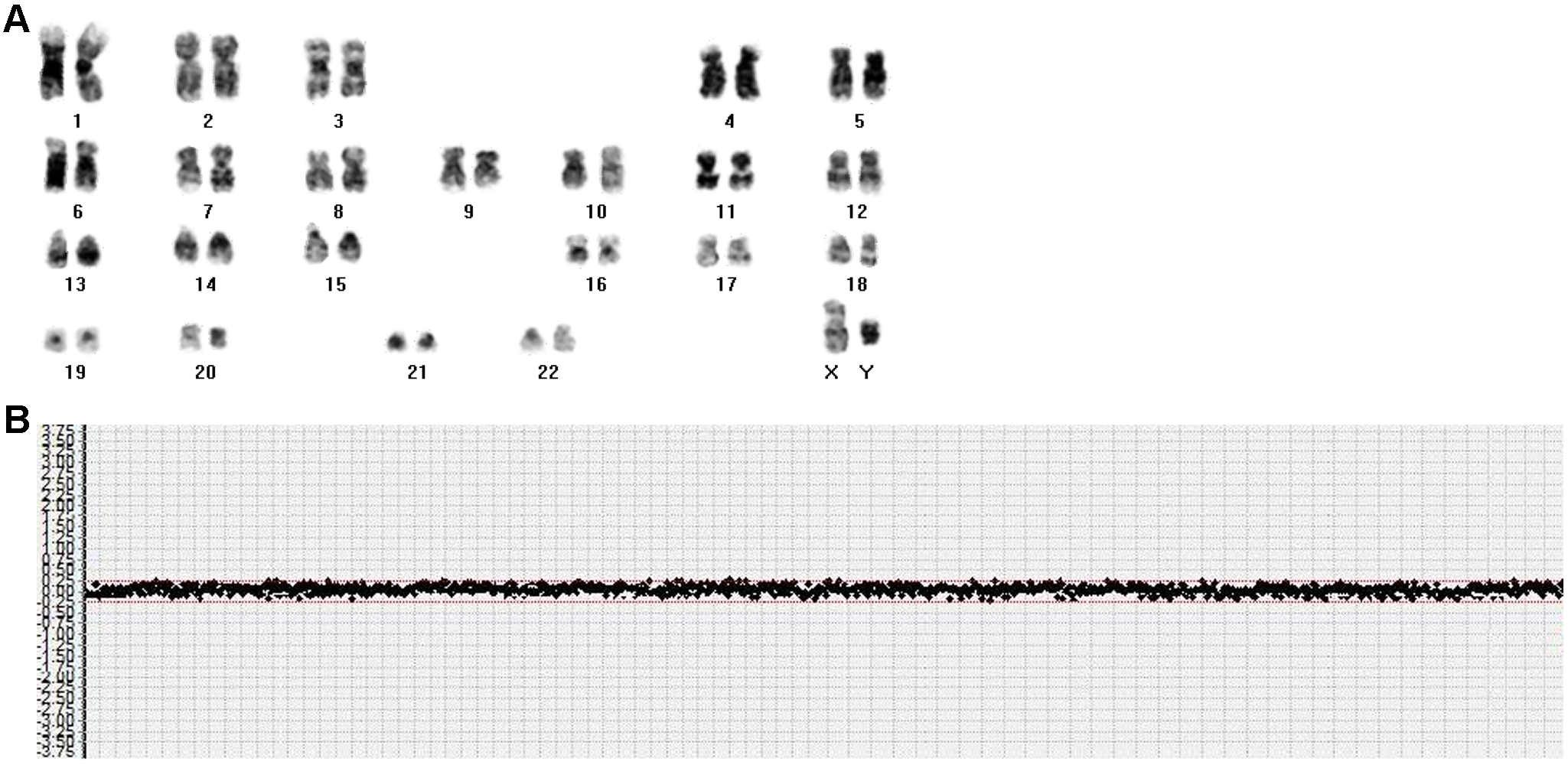

Supplement: Figure S5 — Cytogenetic analysis of human NSPCs. (A) Karyotyping and G-banding analysis of huNSPCs revealed a normal diploid karyotype at passage number 27 (46, XY). (B) Array CGH analysis of huNSPCs further confirmed their normal karyotype. (TIF) [file pone.0104092.s005.tif]
