# Supplementary material for: Human Fetal Brain-Derived Neural Stem/Progenitor Cells Grafted into the Adult Epileptic Brain Restrain Seizures in Rat Models of Temporal Lobe Epilepsy
Source: PLoS One. 2014 Aug 8;9(8):e104092. doi: 10.1371/journal.pone.0104092 (PMC4126719; doi:10.1371/journal.pone.0104092)
Supplement: Table S1 — Primers used for quantitative real-time PCR. (DOCX) [file pone.0104092.s006.docx]

**Table S1.** Primers used for qRT-PCR

| **Gene** | **Strand** | **Sequence (5’ → 3’)** | **Amplicon length (bp)** |
| --- | --- | --- | --- |
| FOXG1 | F | AGAAGAACGGCAAGTACGAGA | 189 |
|  | R | TGTTGAGGGACAGATTGTGGC |  |
| OLIG2 | F | ATGCACGACCTCAACATCG | 141 |
|  | R | CTCCAGCGAGTTGGTGAGC |  |
| ASCL1 | F | CCCAAGCAAGTCAAGCGACA | 77 |
|  | R | AAGCCGCTGAAGTTGAGCC |  |
| DLX2 | F | GCCTCAACAACGTCCCTTACT | 150 |
|  | R | TCACTATCCGAATTTCAGGCTCA |  |
| NKX2.1 | F | AGACTCGCTCGCTCATTTGT | 231 |
|  | R | CTCCATGCCCACTTTCTTGT |  |
| LHX6 | F | GGGCGCGTCATAAAAAGCAC | 108 |
|  | R | TGAACGGGGTGTAGTGGATG |  |
| NR2F2 | F | CGGGTGGTCGCCTTTATGG | 129 |
|  | R | ACAGGCATCTGAGGTGAACA |  |
| GAD1 | F | CGAGGACTCTGGACAGTAGAGG | 182 |
|  | R | GATCTTGAGCCCCAGTTTTCTG |  |
| SLC32A1 | F | ACGTCCGTGTCCAACAAGTC | 117 |
|  | R | AAAGTCGAGGTCGTCGCAATG |  |
| SLC6A1 | F | CCTGGGCCAGTACACCTC | 89 |
|  | R | GCCAGAATGATAGCACAGCA |  |
| CALB2 | F | TCAGAGATGTCCCGACTCCTG | 120 |
|  | R | GCCGCTTCTATCCTTGTCGTAA |  |
| SST | F | GCTGCTGTCTGAACCCAAC | 138 |
|  | R | CGTTCTCGGGGTGCCATAG |  |
| NPY | F | CGCTGCGACACTACATCAAC | 62 |
|  | R | CTCTGGGCTGGATCGTTTTCC |  |
